# Cation Channel TMEM63A Autonomously Facilitates Oligodendrocyte Differentiation at an Early Stage

**DOI:** 10.1007/s12264-024-01338-4

**Published:** 2025-02-21

**Authors:** Yue-Ying Wang, Dan Wu, Yongkun Zhan, Fei Li, Yan-Yu Zang, Xiao-Yu Teng, Linlin Zhang, Gui-Fang Duan, He Wang, Rong Xu, Guiquan Chen, Yun Xu, Jian-Jun Yang, Yongguo Yu, Yun Stone Shi

**Affiliations:** 1 Ministry of Education Key Laboratory of Model Animal for Disease Study, Model Animal Research Center, Department of Neurology, Nanjing Drum Tower Hospital, Medical School, Nanjing University, Nanjing, 210032 China; 2 Department of Anesthesiology, Pain and Perioperative Medicine, First Affiliated Hospital of Zhengzhou University, Zhengzhou, 450052 China; 3 Guangdong Institute of Intelligence Science and Technology, Hengqin, Zhuhai, 519031 China; 4 Center of Clinical Genetics, School of Medicine, Xinhua Hospital Affiliated to Shanghai Jiao Tong University , Shanghai, 200092 China; 5 Shanghai Institute for Pediatric Research, Shanghai, 200092 China; 6 Department of Developmental and Behavioral Pediatrics, Department of Child Primary Care, Brain and Behavioral Research Unit of Shanghai Institute for Pediatric Research and MOE-Shanghai Key Laboratory for Children’s Environmental Health, Xinhua Hospital Affiliated to Shanghai Jiao Tong University School of Medicine, Shanghai, 200092 China; 7 Qilu Pharmaceutical Co., Ltd, Jinan, 250100 China; 8 State Key Laboratory of Natural and Biomimetic Drugs, School of Pharmaceutical Sciences, Peking University, Beijing, 100091 China; 9 Department of Rehabilitation Medicine, Medical School, Nanjing Drum Tower Hospital, Nanjing University, Nanjing, 210008 China; 10 Department of Pediatric Endocrinology and Genetic Metabolism, Xin Hua Hospital Affiliated to Shanghai Jiao Tong University School of Medicine, Shanghai Institute for Pediatric Research, Shanghai, 200092 China

**Keywords:** TMEM63A, Mutation, Oligodendrocyte differentiation, Hypomyelination

## Abstract

Accurate timing of myelination is crucial for the proper functioning of the central nervous system. Here, we identified a *de novo* heterozygous mutation in *TMEM63A* (c.1894G>A; p. Ala632Thr) in a 7-year-old boy exhibiting hypomyelination. A Ca^2+^ influx assay suggested that this is a loss-of-function mutation. To explore how TMEM63A deficiency causes hypomyelination, we generated *Tmem63a* knockout mice. Genetic deletion of TMEM63A resulted in hypomyelination at postnatal day 14 (P14) arising from impaired differentiation of oligodendrocyte precursor cells (OPCs). Notably, the myelin dysplasia was transient, returning to normal levels by P28. Primary cultures of *Tmem63a*^−/−^ OPCs presented delayed differentiation. Lentivirus-based expression of TMEM63A but not TMEM63A_A632T rescued the differentiation of *Tmem63a*^−/−^ OPCs *in vitro* and myelination in *Tmem63a*^−/−^ mice. These data thus support the conclusion that the mutation in TMEM63A is the pathogenesis of the hypomyelination in the patient. Our study further demonstrated that TMEM63A-mediated Ca^2+^ influx plays critical roles in the early development of myelin and oligodendrocyte differentiation.

**Supplementary Information:**

The online version contains supplementary material available at 10.1007/s12264-024-01338-4.

## Introduction

In higher vertebrates, proper myelination of axons is critical for the rapid and accurate transmission of saltatory impulses [1]. Oligodendrocytes (OLs) are the myelinating cells in the central nervous system (CNS); they arise after a series of developmental processes from oligodendrocyte precursor cells (OPCs) to immature OLs and finally to mature OLs [2–4]. Mature OLs send processes to wrap axons, forming the myelin sheath [4, 5]. This intact myelin sheath is essential for neurons to effectively propagate action potentials in a saltatory mode [6]. The processes of OL differentiation and myelination are regulated by various intrinsic pathways and external signals [7–9].

Transmembrane protein 63s (TMEM63s) have recently been discovered to be mechanosensitive cation channels [10–12]. These channels respond to osmolality changes; both hypoosmotic and hyperosmotic stimuli activate recombinant TMEM63 channels, with a particular preference for hypoosmotic stress [13, 14]. The physiological functions of channels in the TMEM63 family have recently been studied. Li et al. revealed that TMEM63 acts as a humidity sensor in *Drosophila* that detects humidity by transforming it into a mechanical stimulus [15]. Li and Montell found that flies discriminate particle sizes in food through the TMEM63 channels on their tongues [16]. In mammals, there are three TMEM63 homologs, TMEM63A–C. Our recent studies found that TMEM63B serves as an osmosensor in the inner ear required for hearing [14] and in the subfornical organ for detecting thirst information [17]. TMEM63B may also regulate thyroid hormone secretion [18]. The splicing and the editing regulate the Ca^2+^ permeability and osmosensitivity of TMEM63B in a coordinated manner [19, 20]. TMEM63A and TMEM63B are required for surfactant secretion in the lung [21]. Tabara et al. associated TMEM63C with morphological defects in the mitochondria and endoplasmic reticulum [22], while Schulz et al. showed that TMEM63C is expressed in the kidney and associated with podocyte function [23]. Pu et al. found that TMEM63A is expressed in non-peptidergic nociceptors in dorsal root ganglia and elevated in local macrophages during chronic post‑amputation pain [24]. TMEM63A protects the pancreas by preventing uncontrolled zymogen secretion in acinar cells [25]. Li et al. provided evidence that *Drosophila* TMEM63 and mouse TMEM63A are located in lysosomes [26]. Cryo-EM studies showed the structures of TMEM63A/B/C are monomers [11, 12, 27, 28] while biochemical evidence indicated that dimers might exist for the TMEM63B channel [20, 28]. Recently, several studies have shown that missense mutations in the *TMEM63A* gene (MIM * 618685) result in a disorder called hypomyelination leukodystrophy (MIM # 618688), in which congenital nystagmus and deficient myelination are displayed in the infant brain [29–32]. However, the underlying mechanisms of TMEM63A-related hypomyelination remain unknown.

TMEM63A is a cation channel that can mediate Ca^2+^ entry [14]. As a universal second messenger, Ca^2+^ is known to regulate a wide range of cellular functions. Recent studies have revealed an important role of Ca^2+^ signaling in OL development in the CNS [33–35], including OPC proliferation [36], OPC migration [37, 38], OL differentiation [39], and initiation of myelination [40, 41]. Moreover, Ca^2+^ transients in OLs also regulate the retraction and elongation of the developing myelin sheath [42–44]. Recent evidence has shown that dynamic changes in Ca^2+^ are associated with oxidative stress and impairments in myelin structure [45–47]. While the importance of Ca^2+^ channels in OL development and myelination is acknowledged, their specific roles remain largely unexplored [48].

In the current study, we identified a patient carrying a mutation in TMEM63A (c.1894G>A; p. Ala632Thr), who displayed developmental delay and hypomyelination. Functional assay indicated that this is a loss-of-function (LoF) mutation. We thus generated *Tmem63a* knockout (*Tmem63a*^*−/−*^) mice to understand how TMEM63A deficiency affects myelination. We found that TMEM63A is abundantly expressed in OL lineage cells. OL differentiation in the postnatal brain is impaired in *Tmem63a*^*−/−*^ mice. Our *in vitro* analysis revealed that loss of TMEM63A disrupts hypoosmolarity-induced Ca^2+^ influx in cultured OLs. Overall, our results highlight a critical role for TMEM63A-mediated Ca^2+^ influx in OL differentiation, providing insights into the disease mechanics for *TMEM63A* mutation-related dysmyelination.

## Materials and Methods

### Ethics

Written informed consent was given by the parents for the purposes of research participation and publishing. The study was conducted in accordance with the Helsinki Declaration and was approved by the Ethics Committee of Xinhua Hospital, School of Medicine, Shanghai Jiao Tong University (No. XHEC-D-2023-218).

### Genetic Testing

Exome sequencing was carried out using the xGen Exome Research Panel v1.0 capture probe (1056114, IDT, Coralville, USA) in accordance with the manufacturer’s instructions. The captured DNA fragments were sequenced on an Illumina HiSeq 4,000 platform (San Diego, USA). Variant calling was carried out following the Genome Analysis Toolkit (GATK) recommended practices (version 3). SnpEff (v4.2) was used to annotate the output variants in the Variant Call Format (VCF). Variants with high frequency in population datasets were excluded. Sanger sequencing was applied to confirm the presence and co-segregation of the candidate variant using the following primers: TMEM63A_1894 forward 5ʹ-CCACGCATCTCCTATTTCCAA-3ʹ and reverse 5ʹ- CCCCGAGAGGTTTGGTGC-3ʹ.

### Mice

*Tmem63a* knockout (*Tmem63a*^*−/−*^) mice were generated based on the clustered regularly interspaced short palindromic repeats (CRISPR)-Cas9 technique [49, 50]. A single guide RNA (sgRNA) targeting exon 2 after the initial codon of mouse *Tmem63a* in company with the purified Cas9 protein was injected into mouse embryos on the C57BL/6N genetic background. A mouse founder carrying a 452-bp fragment deletion from 249 bp to 700 bp after the initial codon, resulting in a reading frameshift, was chosen for further breeding and subsequent experiments.

*Tmem63a*^*EGFP*^ knock-in mice were also generated using CRISPR-Cas9 genome-editing technology. An sgRNA targeting exon 2 after the initial codon of mouse *Tmem63a* in company with the purified Cas9 protein was injected into mouse embryos on the C57BL/6N genetic background to insert the LoxP-EGFP-PolyA-LoxP cassette before the ATG codon of the *Tmem63a* locus, allowing EGFP expression under the driving of the *Tmem63a* promotor. The positive recombinant founder mice were identified by genotyping (E005-01, Novoprotein, Shanghai, China).

Mice were housed in a specific-pathogen-free facility under a 12 h light-dark cycle with free access to food and water. Both male and female animals ranging in age from 7 to 28 days were used. All animal experiments were conducted in accordance with a protocol that followed institutional guidelines and was approved by the Institutional Animal Care and Use Committee of the Model Animal Research Center of Nanjing University.

### Cell Culture

Neuro2a (N2a) mouse neuroblastoma cells were cultured in Dulbecco’s Modified Eagle’s Medium supplemented with 10% fetal bovine serum (C0235, Gibco, Grand Island, USA). The cells were seeded onto poly-D-lysine-coated 8-mm square glass coverslips and placed in 35 mm dishes. The complementary DNA (cDNA) constructs were transfected into the N2a cells using lipofectamine 2,000 transfection reagent (11668019, Invitrogen, Carlsbad, USA) following the manufacturer’s instructions. Human TMEM63A cDNA was cloned into the pCAGGs-P2A-GCaMP6f vector. The A632T mutation was introduced through overlapping PCR. All plasmids were transfected at a constant concentration of 1000 ng/mL.

### Western Blotting

The proteins from each sample were lysed in cold radio-immunoprecipitation assay (RIPA) lysis buffer (PC101, EpiZyme, Shanghai, China) supplemented with a mixture of protease inhibitors (P1005, Beyotime, Shanghai, China). Subsequently, the protein samples were loaded onto 12.5% SDS-PAGE gels (PG113, EpiZyme) and transferred to polyvinylidene fluoride (PVDF) membranes (ISEQ00010, Millipore, Burlington, USA). After blocking with 5% non-fat milk powder in Tris-buffered saline (TBS) containing 0.1% Tween-20 for 1 h, the membranes were incubated overnight at 4 °C with primary antibodies. After three washes, the membranes were incubated with horseradish peroxidase (HRP)-conjugated secondary antibodies and subsequently analyzed using a Chemiluminescent Imaging System (5200, Tanon, Shanghai, China).

### Tissue Preparation and Immunohistochemistry

After perfusion with phosphate-buffered saline (PBS), the brain was removed and fixed in 4% paraformaldehyde for 4 h at 4 °C. Subsequently, all tissues were dehydrated in 30% sucrose for 48 h at 4 °C and then embedded in the optimal cutting temperature (O.C.T) compound (3801481, Leica, Wetzlar, Germany). For immunohistochemistry analysis, sections were blocked with 10% normal goat serum for 45 min at room temperature (RT), followed by overnight incubation with primary antibodies at 4 °C. Following PBS washes, sections were incubated with secondary antibodies conjugated with Alexa Fluor dyes 488/549/647 (Bioworld, Nanjing, China) for 1 h at RT and washed three times in PBS. Nuclei were counterstained with DAPI (1 μg/mL; D9542, Sigma-Aldrich, Saint Louis, USA) before mounting in the mounting medium. Immunofluorescence images were acquired on a confocal laser microscope (LSM880, Zeiss, Jena, Germany). All antibodies used in this study are listed in Table 1.

**Table 1:** List of antibodies used in this study

Reagent	Source	Identifier
Rat anti-Mbp	Millipore	Cat#MAB386
Rabbit anti-Mag	Abcam	Cat#ab277524,
Mouse anti-Olig2	Millipore	Cat#MABN50
Mouse anti-CC1	Calbiochem	Cat#op-80
Rabbit anti-PDGFRα	Abcam	Cat#ab203491
Rabbit anti-Ki67	Abcam	Cat#ab15580
Rat anti-BrdU	Abcam	Cat#ab6326
Rabbit anti-β-Tubulin	Bioworld	Cat#AP0060
Chicken anti-GFP	Abcam	Cat#13970
Rabbit Cleaved caspase-3 (ASP175)	Cell Signaling Technology	Cat#9661

### TrueGold Myelin Staining

Brains were sectioned at 20 μm as above. The sections were washed with distilled water and then incubated with TrueGold myelin solution (BK-AC001, Oasis Biofarm, Hangzhou, China) for 30 min at 45°C. Sections were then washed with distilled water and cover-slipped with mounting medium.

### Transmission Electron Microscopy (TEM)

The tissue was prepared according to previously described methods [51]. Briefly, P14 and P28 mice were anesthetized and perfused with pre-chilled sodium cacodylate buffer followed by 2.5% glutaraldehyde. The cortex was immediately dissected out, placed in Petri dishes (1140040, SAINING Biotechnology, Taizhou, China), and cut into 1 mm^3^ pieces in the fixative. Subsequently, the 1 mm^3^ blocks were fixed overnight at 4 °C in 2.5% glutaraldehyde. Finally, the prepared tissues were transferred to Servicebio Technology Co., Ltd. (Wuhan, China) for further processing.

### Bromodeoxyuridine (BrdU) Assay

To study OPC proliferation, BrdU (B5002, Sigma–Aldrich) was intraperitoneally injected into mice at 100 mg/kg for 2 h at P4. Sections were then prepared and used for double-staining of BrdU/Olig2. To study OL differentiation, BrdU was intraperitoneally injected into mice at 100 mg/kg for 6 consecutive days from P4 to P9. Brain sections were then prepared at P14 and used for double-staining of BrdU/CC1.

### Cell Counting

The brain sections, spaced at 20 μm intervals, were used for immunofluorescence analysis. Five mice per genotype were included in the cell-counting experiments. Images of the cortex were subjected to area measurement and cell counting using ImageJ software. For statistical analysis, images were acquired under either a 10× or 20× objective on a Zeiss LSM880 microscope.

### Primary OPC Cultures

A method described previously [52, 53] was used with slight modifications. The cortex of neonatal mice at P7 was carefully dissected and gently triturated, followed by filtration through a 40-mm filter to obtain a single-cell suspension. Cells were plated into T25 culture flasks (707003, NEST, Wuxi, China) in serum-free conditioned DMEM/F12 medium (11330-032, Gibco) (oligosphere medium) containing 1% N2 (17502048, Invitrogen), 20 µg/mL insulin (I6634, Sigma-Aldrich), 2% B-27 (17504044, Invitrogen), 10 ng/mL PDGF-AA (100-13A, PeproTech, Rocky Hill, USA), 10 ng/mL bFGF (100-18B, PeproTech), 5 mmol/L HEPES (15630-080, Gibco) and 100× GlutaMAX (35050-061, Gibco). Three days later, OPCs were induced to exit from the cell cycle and differentiate by switching to a mitogen-free medium: DMEM/F12 supplemented with 2% B-27, 1% N2, and 15 nmol/L T3 (T-2752, Sigma).

### Cytoplasmic Ca^2+^ Measurements

Cytoplasmic Ca^2+^ in primary cultured OLs was measured as previously reported [54, 55]. In brief, OPCs cultured on coverslips were incubated with 4 mmol/L Fluo-4-AM plus 0.05% Pluronic F127 (P2443, Sigma-Aldrich) for 30 min at 37 °C, then washed with DMEM and transferred to isotonic extracellular solution (in mmol/L): 65 NaCl, 5 KCl, 1 CaCl_2_, 1 MgCl_2_, 10 HEPES (pH 7.4 adjusted with NaOH, 300 mOsm/L adjusted with mannitol), for imaging. The isotonic solution was exchanged for 170 mOsm/L hypotonic solution by a peristaltic pump (BT100-2J, LONGER, Baoding, China) at a constant speed without changing the ionic concentrations. The osmolarity of solutions was measured by a vapor pressure osmometer (Vapro 5600, Wescor, Logan, USA). Ca^2+^ fluorescence was recorded at room temperature (24 ± 2 °C) at 1 Hz for 13 min by a Hamamatsu digital imaging camera (C11440-22U, Hamamatsu, Shizuoka, Japan) using 488-nm illumination. More than 80 cells for each experimental condition were analyzed and the results from three separate experiments were pooled. To minimize bleaching, the intensity of excitation light and sampling frequency were kept as low as possible. The change of fluorescence was normalized by the ratio of real-time intensity (*F*_t_) relative to the initial value (*F*_0_). The cells with *F*_t_/*F*_0_ >1.5 were considered to show positive responses to the hypotonic challenge.

For N2a cells, the cytoplasmic Ca^2+^ concentration was monitored using the free Ca^2+^ indicator GCaMP6f [14]. TMEM63A-P2A-GCaMP6f vectors were transfected into the N2a cells cultured on a coverslip. After 40 h of transfection, Ca^2+^ fluorescence was examined while the cells were perfused with extracellular solutions of varying osmolarity at a constant exchange rate using a peristaltic pump.

### Lentivirus Infection

The pGC-CMV-TMEM63A-GFP-lentivirus and pGC-CMV-TMEM63A_A632T-GFP-lentivirus (GeneChem, Shanghai, China) were each added to OPCs seeded on 6-well plates. OPCs were kept in the oligosphere medium with lentivirus (LV) for 72 h and then were differentiated by switching the cells to a mitogen-free medium for three days. The GFP gene expression was observed under the fluorescence microscope and the cells were collected for subsequent culture. Expression of Mbp was analyzed by immunohistochemistry (IHC).

### Stereotaxic Surgery

At P1, mice were anesthetized with isoflurane and placed into a stereotaxic frame. Lentivirus was bilaterally injected into the cortex through a beveled glass micropipette using 10 nL of viral preparation per hemisphere. Three injection sites per hemisphere were used to optimize virus spread. The injection coordinates for the cortex were as follows (in mm): −2.5 from lambda; lateral ±1.4; ventral, 0.4/0.2/0.1. Viral-mediated protein expression was allowed for 13 days before experimental manipulation.

### Statistical Analysis

The data are presented as the mean ± SEM. Statistical significance was defined as *P* <0.05. Two-tailed Student’s *t*-test was applied to evaluate the difference between control and *Tmem63a*^*−/−*^ mice. 3–6 mice in each genotype were used for analysis.

## Results

### Clinical Findings in Proband with TMEM63A_A632T Mutation

**Fig. 1:**
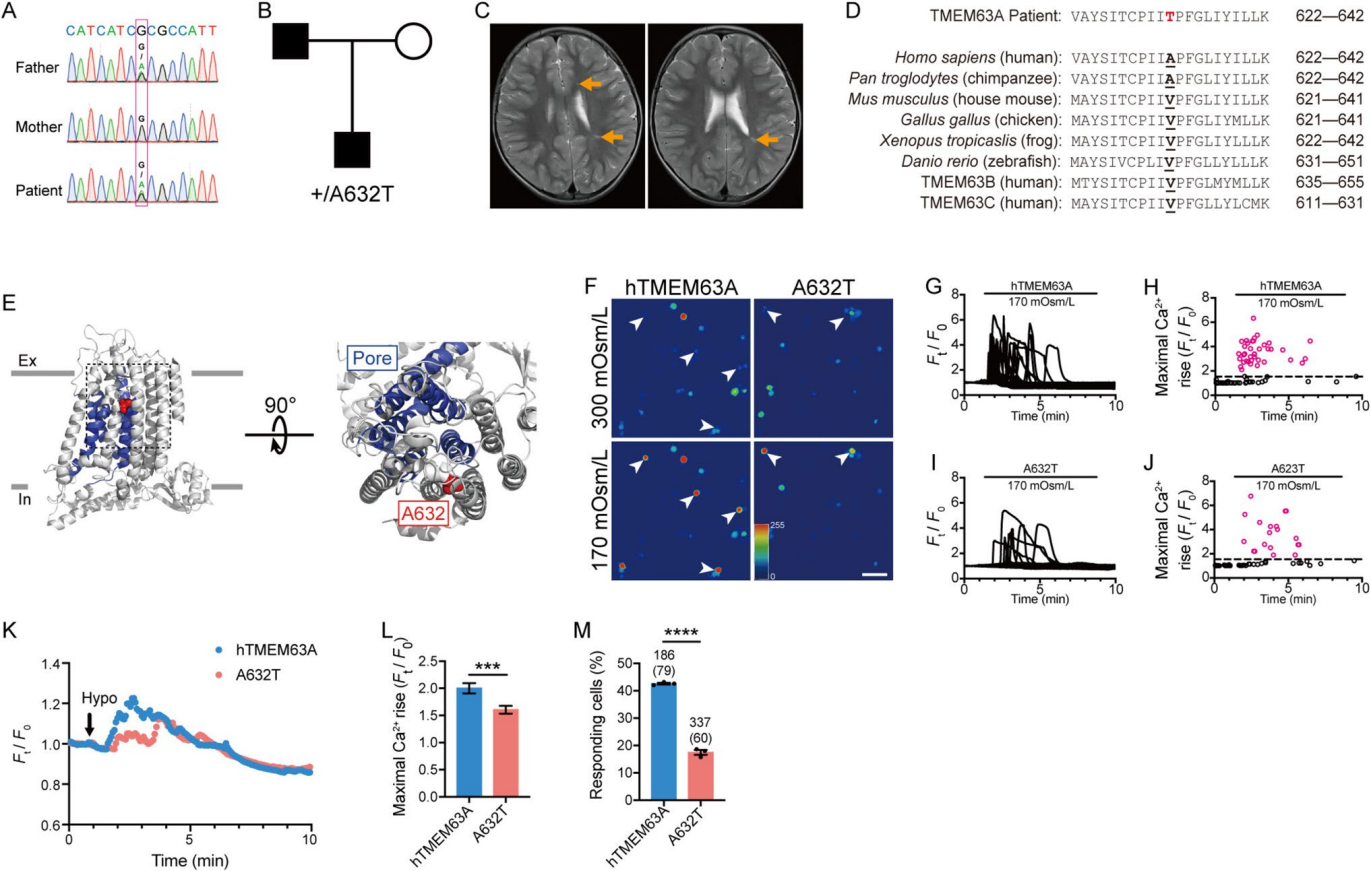
TMEM63A_A632T is a loss-of-function mutation. **A** Sanger sequencing around the mutant site in the proband and his parents. **B** Family pedigree of the rare variant. Black symbols represent individuals carrying the rare variant, while white symbols represent individuals without the variant. The circle denotes women, and the squares denote men. **C** MRIs (axial T2-weighted images) of the patient’s brain, which showed abnormal signals in the left ventricular posterior horn (orange arrows). **D** Sequence alignment of TMEM63A around A632 among species. **E** Structure of TMEM63A protein based on Zheng, et al. [11] (Protein Data Bank: 8ehw) and the location of A632 (spheres). The extracellular (Ex) and intracellular (In) surfaces of the plasma membrane are indicated by gray lines. The pore-lining transmembrane helices are shown in blue. **F** Fluorescent emission images of GCaMP6f-positive cells in response to 170 mOsm/L hypotonic solution. White arrowheads indicate the responding cells. Scale bar, 100 µm. **G** Ca^2+^ fluorescence intensity traces of cells expressing TMEM63A-P2A-GCaMP6f responding to 170 mOsm/L solution in a representative experiment. **H** The maximal responses of cells pooled from three experiments challenged by 170 mOsm/L solution plotted *versus* the exposure time. Red, Ca^2+^ fluorescence increases >50% are considered to be positively-responding cells. **I** Ca^2+^ fluorescence intensity traces of cells expressing TMEM63A_A632T-P2A-GCaMP6f in response to 170 mOsm/L solution in a representative experiment. **J** The maximal responses of cells pooled from three experiments challenged by 170 mOsm/L solution *versus* the exposure time. **K** Averaged Ca^2+^ fluorescence changes upon hypotonic challenge (Hypo). **L** Statistical analysis of the maximal Ca^2+^ rise in response to 170 mOsm/L solution. Cells transfected with TMEM63A_A632T have a lower Ca^2+^ transient (***P <0.001, *t*-test; hTMEM63A: *n* = 186 cells; A632T: *n* = 337 cells). **M** Percentage of cells responding to 170 mOsm/L solution. The responding ratio is significantly reduced in A632T compared with WT TMEM63A (****P <0.0001,* t*-test; *n* = 3 experiments). The number of cells tested and responding (in brackets) is indicated above the bars.

The proband is a 7-year-old boy (Fig. 1A, B). At 4 years old, he exhibited developmental delay, particularly in fine motor skills and expressive language. In addition, he displayed impaired social interaction, stereotypic repetitive behaviors, and hyperactivity, and was diagnosed with autistic spectrum disorder (ASD) and attention deficit hyperactivity disorder (ADHD). Brain magnetic resonance imaging (MRI) revealed a severe deficit in myelin, with a low-normal myelin signal in the left ventricular posterior horn in T2-weighted images (Fig. 1C). By the age of 5, there was an improvement in his linguistic competence. However, in school, he exhibited social difficulties, accompanied by behavioral issues such as sudden yelling and disobeying instructions. At 7 years old, ASD and ADHD persisted.

To explore possible causal genetic variants, we conducted exome sequencing (ES) on the individual, revealing a heterozygous missense variant of *TMEM63A* (GenBank: NM_014698.3; c.1894G>A, p.[Ala632Thr]). In the general population of ExAC and gnomAD databases, the allele frequency of the c.1894G>A variant is 0.0008249% and 0.001591% respectively. Notably, this variant was conspicuously absent in controls of East Asian descent within both databases. The affected amino-acid position, p.Ala632, is conserved (positive scores in PhyloP: 4.077). The missense variant p.Ala632Thr was predicted to be damaging by the functional prediction algorithms combined annotation-dependent depletion (CADD) (Phred Score = 28.7), deleterious annotation of genetic variants using neural networks (DANN) (Score: 28.7), and likelihood ratio test (LRT) (Score: 0.00). No other clinically relevant deleterious mutations were found.

Sanger sequencing was applied to analyze the locus for the parents, revealing that the rare variant in the proband was inherited from his father (Fig. 1A, B). Notably, the father displayed a sluggish response in verbal communication and disclosed a history of delayed language development and limited verbal interactions during his youth. This background information suggested the possibility of developmental delay, particularly in language, for the father. This comprehensive analysis led us to hypothesize that the heterozygous mutation in TMEM63A is most likely responsible for the myelin sheath abnormality in the patient. This variant impacts an amino-acid situated within the transmembrane domain (M8) (Fig. 1D). The residue is alanine in the human and chimpanzee, but valine in other animals including the mouse, chicken, frog, and zebrafish. Interestingly, valine is also present in human TMEM63B and TMEM63C. Despite the evolutionary transition from valine to alanine in some mammals, both valine and alanine share common characteristics as hydrophobic residues with small side chains. The patient’s mutation introduces a hydrophilic residue, threonine, in place of the original alanine, potentially disrupting the channel function (Fig. 1E) [11].

### TMEM63A_A632T Is a Loss-of-function Mutation

To study the effect of this mutation on TMEM63A function, we cloned human TMEM63A and constructed TMEM63A-P2A-GCaMP6f in the pCAGGs vector. The construct was transfected into N2a cells, allowing separate expression of TMEM63A and the Ca^2+^-sensitive reporter GCaMP6f. A hypotonic solution (170 mOsm/kg) was applied to activate TMEM63A and Ca^2+^ fluorescence was monitored (Fig. 1F) [14]. The [Ca^2+^]_i_ elevation was significantly lower in N2a cells transfected with TMEM63A_A632T than in those with wild-type TMEM63A (Fig. 1G–L). In addition, the ratio of responding cells among the total population was markedly reduced (Fig. 1M). These findings strongly suggest that the A632T variant represents a LoF mutation in TMEM63A.

### TMEM63A Is Abundantly Expressed in OLs in the Brain

**Fig. 2:**
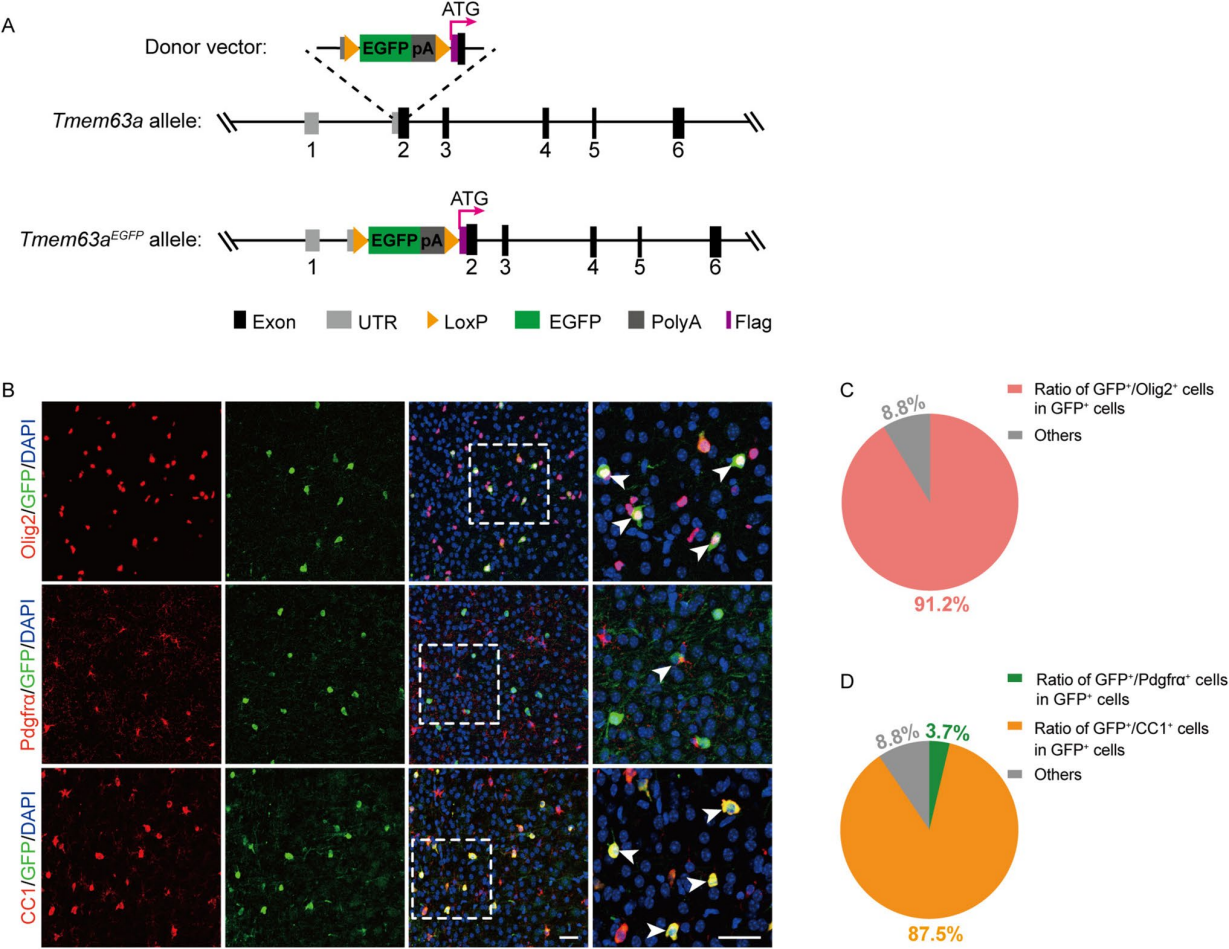
TMEM63A is abundantly expressed in oligodendrocytes in the brain. **A** Generation of *Tmem63a*^*EGFP*^ mice. **B** Co-immunostaining of GFP with Olig2, Pdgfrα, or CC1 in the cortex of *Tmem63a*^*EGFP*^ at P14. The panels on the right are enlarged from the boxed areas. The white arrowheads indicate the double positive cells. Scale bars, 50 μm. **C** Numbers of Olig2^+^ cells among GFP^+^ cells in the cortex at P14. **D** Numbers of CC1^+^ or Pdgfrα^+^ cells among GFP^+^ cells in the cortex at P14.

We then sought to study the function of TMEM63A in myelination. To visualize the expression of TMEM63A in the brain, we generated a *Tmem63a*^*EGFP*^ knock-in reporter mouse line. In this model, a *loxP-EGFP-polyA-loxP* sequence was inserted in front of the start codon of the endogenous *Tmem63a* gene (Fig. 2A). Consequently, EGFP expression was driven by the *Tmem63a* promoter, providing insight into the TMEM63A expression pattern. Double staining for Olig2 and GFP (Fig. 2B) revealed that the majority of GFP^+^ cells in the cortex (~91.2%) were Olig2-positive at P14 (Fig. 2C), indicating predominant expression of TMEM63A in OL lineage cells. Within this population, 3.7% were co-stained with Pdgfrα, a marker of OPCs, while the remaining cells co-stained with CC1, indicating differentiated OLs (Fig. 2D).

We further examined EGFP expression in the brains of *Tmem63a*^*EGFP*^ mice at different developmental stages. Western blots of whole brain lysis showed developmental increases in the EGFP expression in the *Tmem63a*^*EGFP*^ mice. Interestingly, the EGFP expression is nicely coordinated with the expression of Mbp, indicating that TMEM63A expression is increased with OL differentiation and maturation (Fig. S1A, B).

### Loss of TMEM63A Disrupts Proper Myelination

**Fig. 3:**
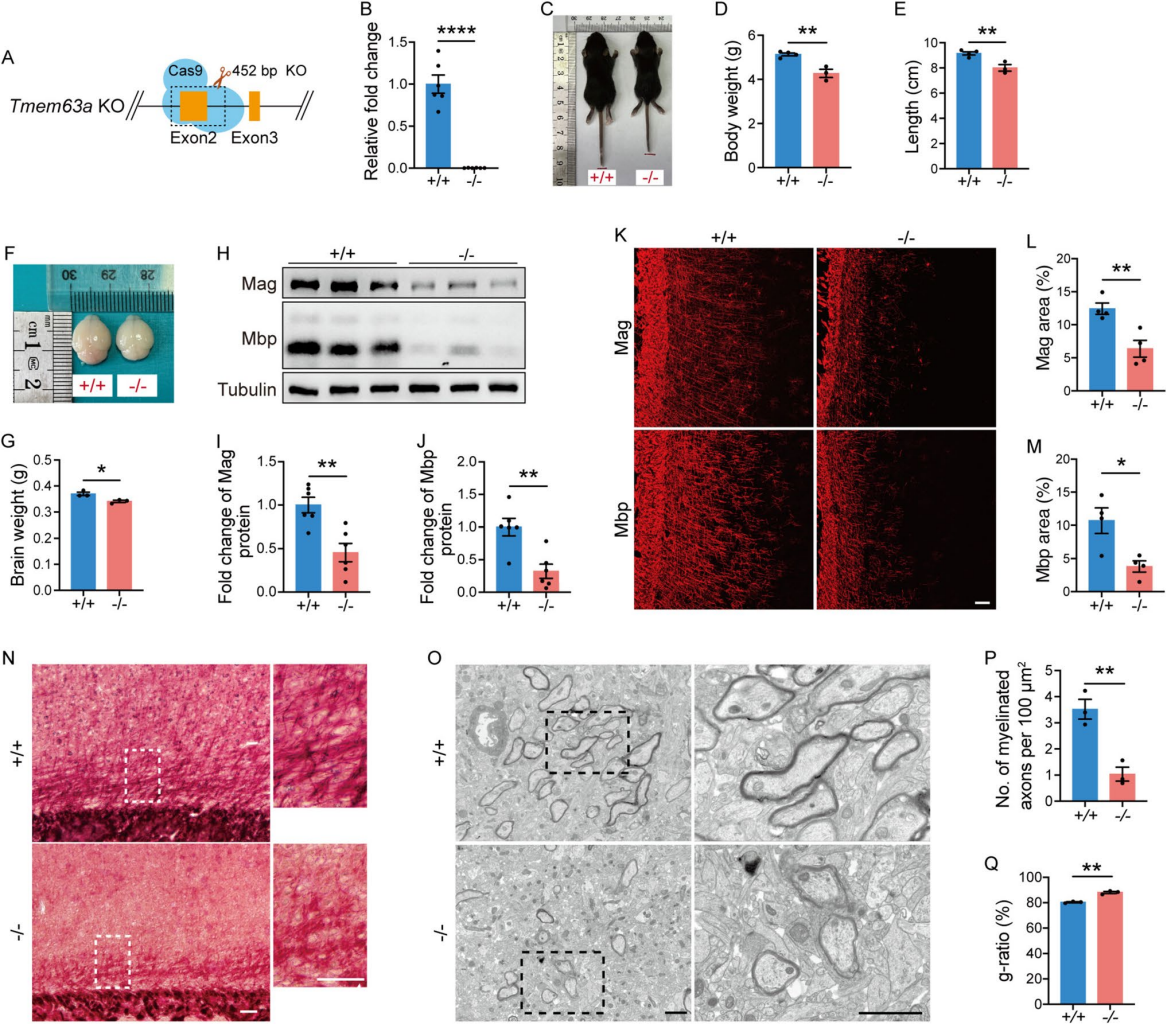
Loss of TMEM63A disrupts proper myelination. **A** Generation of *Tmem63a* knockout mice: Exon2 of TMEM63A is completely deleted. **B** qRT-PCR analyses of *Tmem63a* in the brains of 2-month-old *Tmem63a*^*−/−*^ and WT mice (****P <0.0001, *t*-test; *n* = 6 mice per genotype). **C–E** Body size of WT and *Tmem63a*^*−/−*^ mice (**C**). The body weight (**D**; *P* = 0.0043, *t*-test) and body length (**E**; *P* = 0.0082, *t*-test) are lower in *Tmem63a*^*−/−*^ mice than in WTs at P14 (WT, *n* = 4 mice; *Tmem63a*^*−/−*^, *n* = 3 mice). **F** Whole brains of WT and *Tmem63a*^*−/−*^ mice at P14. **G** Analysis shows a severe reduction in the size of the brain in *Tmem63a*^*−/−*^ mice (*P* = 0.0216, *t*-test; *n* = 3 mice per group). **H** Western blots for Mag and Mbp. Lysates are prepared from the cortex in WT and *Tmem63a*^*−/−*^ mice at P14. β-Tubulin is used as the loading control. **I, J** Fold change in Mag (*P* = 0.0028, *t*-test) and Mbp (*P* = 0.0028, *t*-test) between WT and *Tmem63a*^*−/−*^ mice (*n* = 6 mice per group). **K** Representative images for fluorescence IHC of Mag and Mbp. Scale bar, 100 μm. **L, M** Ratio of the immuno-reactivity area to total area. The immuno-reactivity of Mag (*P* = 0.0074, *t*-test) or Mbp (*P* = 0.0166, *t*-test) in the cortex in the *Tmem63a*^*−/−*^ mice at P14 (*n* = 4 mice per group). **N** TrueGold myelin staining in sections from WT and *Tmem63a*^*−/−*^ mice at P14. The myelin sheath tracts in the cortex are sparse in *Tmem63a*^*−/−*^ mice. The panels on the right are enlarged from the boxed areas. Scale bars, 100 μm. **O** Electron microscopic analysis of the myelin sheath. The panels on the right are enlarged from the boxed areas. Scale bars, 2 μm. **P** The number of myelinated axons per 100 μm^2^ is significantly lower in *Tmem63a*^*−/−*^ mice (*P* = 0.0058, *t*-test; *n* = 3 mice per group). **Q** Quantification of myelin sheath thickness and the bar graph display the g-ratios of all axons as a function of axonal diameter. The *g*-ratio is significantly higher in *Tmem63a*^*−/−*^ mice (*P* = 0.0010, *t*-test; *n* = 3 mice per group). Data are presented as the mean ± SEM and analyzed with unpaired Student’s *t*-test. *P <0.05, **P <0.01.

To investigate the function of TMEM63A in the brain, we used a CRISPR-Cas9-based gene targeting strategy to delete *Tmem63a* in mice (Fig. 3A). No detectable *Tmem63a* mRNA in the brains of 2-month *Tmem63a*^*−/−*^ mice indicated successful deletion of *Tmem63a* (Fig. 3B). At the time of birth, *Tmem63a*^*−/−*^ mice were visually indistinguishable from wild-type littermates (WTs), showing no defects in suckling. However, *Tmem63a*^*−/−*^ mice were notably smaller than WTs at P14 (Fig. 3C). These pups displayed a significant reduction in weight (Fig. 3D) and length (Fig. 3E). Furthermore, the brain size was notably smaller (Fig. 3F, G).

To find out whether the myelin sheath is affected in *Tmem63a*^*−/−*^ mice, we made Western blots of myelin-related proteins using cortical (Fig. 3H) and corpus callosum (Fig. S2A) tissues from P14 mice. The levels of Mag and Mbp proteins were significantly reduced in the cortex (Fig. 3I, J) and corpus callosum (Fig. S2B, C) of *Tmem63a*^*−/−*^ mice compared with WTs. Consistent with this, immunofluorescence (IF) showed significantly decreased Mag and Mbp signals in the cortex (Fig. 3K–M) and corpus callosum (Fig. S2D–F) of *Tmem63a*^*−/−*^ mice. TrueGold myelin staining demonstrated a reduction in positive signals in the brain slices from *Tmem63a*^*−/−*^ mice compared to those from WTs in the cortex (Fig. 3N) and corpus callosum (Fig. S2G). Lastly, we performed an ultra-structural analysis using TEM (Fig. 3O) [3]. The number of myelinated axons was reduced in *Tmem63a*^*−/−*^ mice compared with WTs at P14 (Fig. 3P). In addition, the ratio of the diameter of an axon to the diameter of the axon and myelin sheath (*g*-ratio) was significantly larger in *Tmem63a*^*−/−*^ mice than in WTs (Fig. 3Q), suggesting that the myelin sheath is thinner in *Tmem63a*^*−/−*^ mice. Taken together, the above results suggest that *Tmem63a* deficiency leads to hypomyelination in the brain.

### Loss of TMEM63A Impedes OL Differentiation in the Brain

**Fig. 4:**
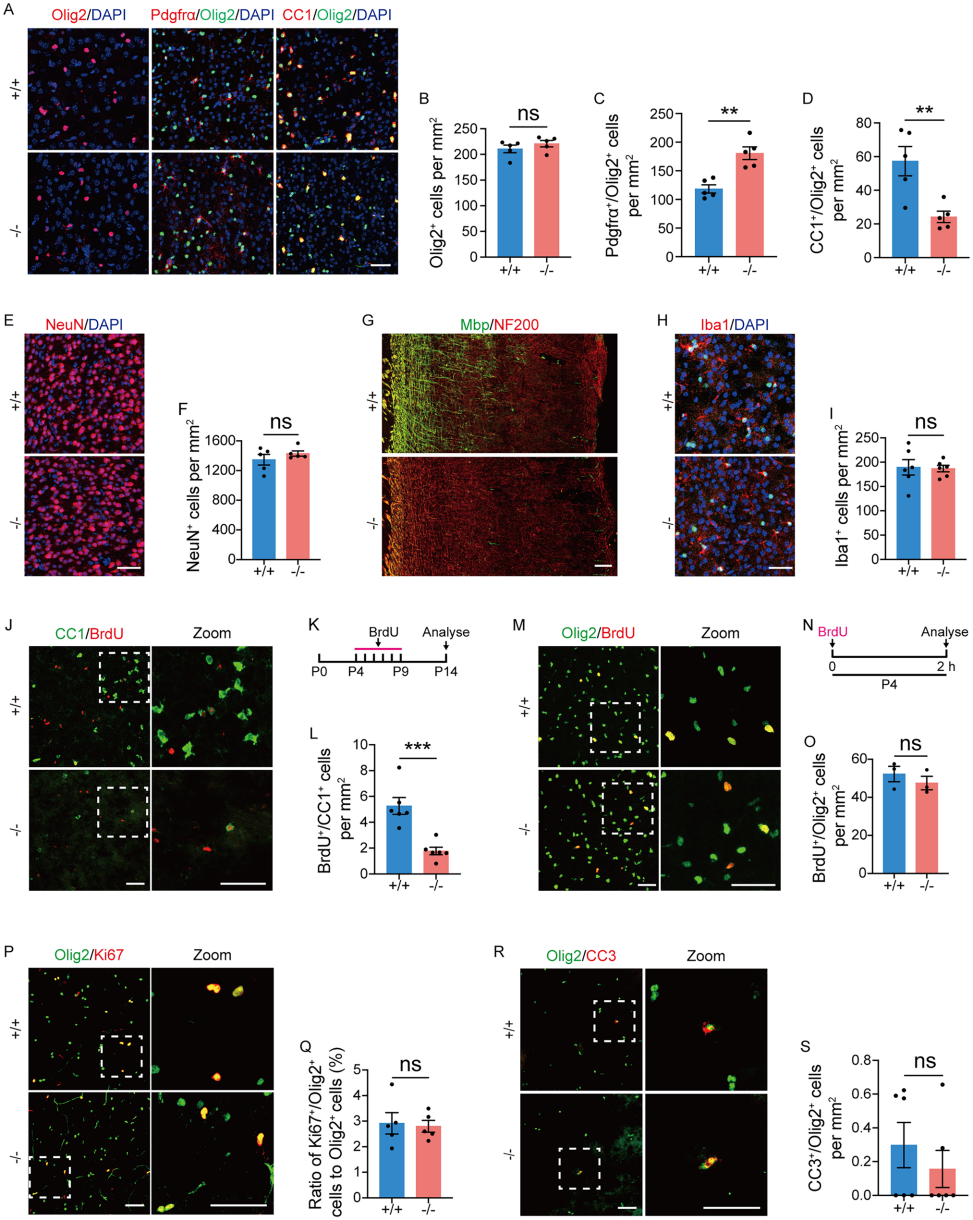
Loss of Tmem63A impedes oligodendrocyte differentiation in the brain. **A** Representative images for IF of Olig2, Pdgfrα/Olig2, and CC1/Olig2. Images are from the cortex of control and *Tmem63a*^*−/−*^ mice at P14. **B** The density of Olig2^+^ cells in the cortex (*P* = 0.3279, *t*-test; *n* = 5 mice per group; ns, no significance). **C** The density of Pdgfrα^+^/Olig2^+^ cells in the cortex (*P* = 0.0015, *t*-test; *n* = 5 mice per group). **D** The density of CC1^+^/Olig2^+^ cells in the cortex (*P* = 0.0075, *t*-test; *n* = 5 mice per group). **E** Representative images for IF on NeuN. Images are from the cortex of control and *Tmem63a*^*-/-*^ mice at P14. **F** The density of NeuN^+^ cells in the cortex (*P* = 0.3304, *t*-test; *n* = 5 mice per group). **G** Representative images for fluorescence IHC on NF200 and Mbp. Scale bar, 100 μm. **H** Representative images for IF on Iba1. Images are from the cortex of control and *Tmem63a*^*-/-*^ mice at P14. **I** The density of Iba1^+^ cells in the cortex (*P* = 0.8770, *t*-test; *n* = 6 mice per group). **J** Representative images of double-staining for BrdU/CC1 in the cortex. The panels on the right are enlarged from the boxed areas. **K** Experimental paradigm for BrdU injection in the cortex of mice. BrdU is injected once a day for six days from P4 to P9. Brain sections are cut at P14. **L** The number of BrdU^+^/CC1^+^cells per mm^2^ in the cortex (*P* = 0.0006, *t*-test; *n* = 6 mice per group). **M** Representative images of double-staining for Olig2/BrdU. The panels on the right are enlarged from the boxed areas. **N** Experimental paradigm for BrdU injection in the cortex of mice at P4. BrdU is injected for 2 h before cutting sections. **O** Numbers of BrdU^+^/Olig2^+^ cells per mm^2^ (*P* = 0.4286, *t*-test; *n* = 3 mice per group). **P** Representative images of double-staining for Olig2/Ki67 in the cortex. Sections from the control and *Tmem63a*^*−/−*^ mice at P14 are used. The panels on the right are enlarged from the boxed areas. **Q** Ratio of Ki67^+^/Olig2^+^ cells to Olig2^+^ cells (*P* = 0.8130, *t*-test; *n* = 5 mice per group). **R** Representative images of double-staining for CC3/Olig2 in the cortex. Sections from the control and *Tmem63a*^*−/−*^ mice at P14 are used. The panels on the right are enlarged from the boxed areas. **S** Number of CC3^+^/Olig2^+^ cells per mm^2^ (*P* = 0.4306, *t*-test; *n* = 6 mice per group). Scale bars, 50 μm in **A**, **E**, **H**, **J, M, P**, and **R,** and 100 μm in** G**. ns, not significant; **P <0.01, ***P <0.001.

To determine the cellular mechanisms underlying the hypomyelination phenotypes in *Tmem63a*^*−/−*^ mice, we analyzed OL lineage cells using brain sections at P14. We found no difference in the density of Olig2^+^ cells in the cortex (Fig. 4A, B) and corpus callosum (Fig. S3A, B) between WT and *Tmem63a*^*−/−*^ mice, suggesting that loss of TMEM63A does not affect the total population of OL lineage cells in the brain. However, the density of Pdgfrα^+^/Olig2^+^ cells in the cortex (Fig. 4A, C) and corpus callosum (Fig. S3A, C) of *Tmem63a*^*−/−*^ mice was higher than that in WTs at P14. In contrast, the density of CC1^+^/Olig2^+^ cells in *Tmem63a*^*−/−*^ mice was significantly lower in the cortex (Fig. 4A, D) and corpus callosum (Fig. S3A, D) than in WTs. To explore whether the neuron or microglia affect myelin development, we performed immunofluorescence staining experiments on NeuN (Fig. 4E) and Iba1 (Fig. 4H). We found that the density of NeuN^+^ cells (Fig. 4F) and Iba1^+^ cells (Fig. 4I) were unaltered in the *Tmem63*^*−/−*^ mice compared with WT mice. Consistent with this, IF on brain sections at P14 indicated that the immunoreactivity of NF200 in *Tmem63a*^*−/−*^ mice was comparable to that in WTs (Fig. 4G**)**. Overall, these results suggest that deletion of TMEM63A does not change the population of neurons or microglia. The reduction of CC1^+^/Olig2^+^ cells and the increase of Pdgfrα^+^/Olig2^+^ cells may be indicative of the failure of OPCs to differentiate into OLs at P14 in *Tmem63a*^*−/−*^ mice. To test this possibility, we performed bromodeoxyuridine (BrdU) birth-dating experiments [3]. BrdU was intraperitoneally injected into mice at P4 for six consecutive days and brain sections were prepared at P14 (Fig. 4K). BrdU^+^/CC1^+^ cells were rarely detected in the cortex (Fig. 4J) and corpus callosum (Fig. S3E) of *Tmem63a*^*−/−*^ mice. Compared to WTs, the density of BrdU^+^/CC1^+^ cells was significantly decreased in the cortex (Fig. 4L) and corpus callosum (Fig. S3F) of *Tmem63a*^*−/−*^ mice. These data demonstrated that OL differentiation is impaired in *Tmem63a*^*−/−*^ mice at P14.

The increase of Pdgfrα^+^/Olig2 ^+^ cells in *Tmem63a*^*−/−*^ mice could also result from enhanced OPC proliferation. To examine this possibility, we injected BrdU into pups at P4 and collected brain sections 2 h later (Fig. 4N). The density of BrdU^+^/Olig2^+^ cells in the cortex (Fig. 4M, O) and corpus callosum (Fig. S3G, H) did not differ between WT and *Tmem63a*^*−/−*^ mice. Furthermore, co-staining with Ki67/Olig2 showed no significant difference in the ratio of Ki67^+^/Olig2^+^ cells to Olig2^+^ cells in the cortex (Fig. 4P, Q) and corpus callosum (Fig. S3I, J) between WT and *Tmem63a*^*−/−*^ mice at P14. These experiments thus demonstrated that deletion of TMEM63A does not affect the proliferation of OPCs in the CNS. The increase of Pdgfrα^+^/Olig2^+^ cells in *Tmem63a*^*−/−*^ mice is more likely to be due to failure to differentiate into OLs.

The reduction of CC1^+^/Olig2^+^ cells may also be caused by the apoptosis of OLs. Thus we studied apoptosis by staining cleaved caspase3 (CC3) using brain sections at P14 (Fig. 4R). There was no difference in the density of CC3^+^/Olig2^+^ cells in the cortex between WT and *Tmem63a*^*−/−*^ mice (Fig. 4S), and CC3^+^/Olig2^+^ was hardly observed in the corpus callosum (Fig. S3K), suggesting that deletion of TMEM63A does not cause abnormal apoptosis in OL lineage cells in the CNS. The decrease of CC1^+^/Olig2 ^+^ cells in *Tmem63a*^*−/−*^ mouse is more likely due to reduced differentiation.

### Myelin in ***Tmem63a***^***−/−***^ Mice Is Normal at P28

**Fig. 5:**
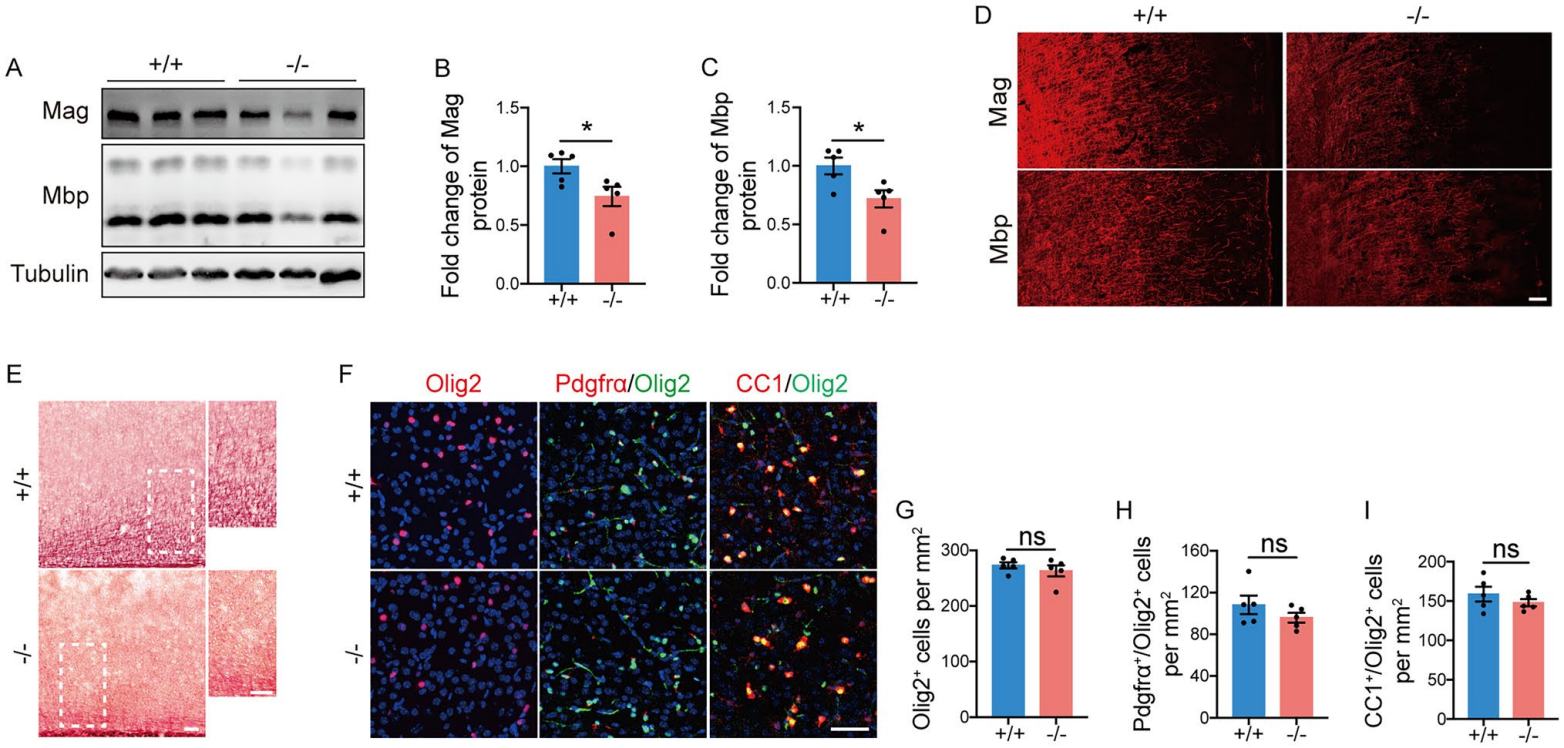
Myelin abnormalities exist in *Tmem63a*^*−/−*^ mice at P21. **A** Western blots for Mag and Mbp. Lysates are prepared from the cortex in WT and *Tmem63a*^*−/−*^ mice at P21. β-Tubulin is used as the loading control. **B, C** Fold change of protein levels for Mag (**B**) (*P =* 0.0359, *t*-test) or Mbp (**C**) (*P =* 0.0254, *t*-test). *n* =5 mice per group. **D** Representative images for fluorescence IHC on Mag and Mbp. Scale bar, 100 μm. The immuno-reactivity on Mag or Mbp is low in the cortex in the *Tmem63a*^*−/−*^ mice at P21. **E** TrueGold myelin staining. Brain sections from WT and *Tmem63a*^*−/−*^ mice at P21 are used. The panels on the right are enlarged from the boxed areas. Scale bars, 100 μm.** F** Representative images for IF on Olig2, Pdgfrα/Olig2, and CC1/Olig2. Images are from the cortex of control and *Tmem63a*^*-/-*^ mice at P21. Scale bar, 50 μm.** G** The density of Olig2^+^ cells in the cortex (*P* = 0.4176, *t*-test; *n* = 5 mice per group). **H** The density of Pdgfrα^+^/Olig2^+^ cells in the cortex (*P* = 0.2569, *t*-test; *n* = 5 mice per group). **I** The density of CC1^+^/Olig2^+^ cells in the cortex (*P* = 0.3247, *t*-test; *n* = 5 mice per group). *P <0.05; ns, not significant.

Several studies have shown that missense mutations in the *TMEM63A* gene result in temporary deficient myelination in the brain of infants [29–31]. To explore whether such hypomyelination is also transient in *Tmem63a*^*−/−*^ mice, we first conducted a Western blotting analysis on myelin-related proteins (Fig. 5A), using cortical tissues from P21 mice. We found that the expression levels of Mag and Mbp remained decreased in the cortex of *Tmem63a*^*−/−*^ mice relative to the WTs (Fig. 5B, C). IF (Fig. 5D) and TrueGold myelin staining (Fig. 5E) confirmed these findings, with fewer myelin sheath fibers in P21 *Tmem63a*^*−/−*^ mice than in WTs. However, there was no difference in the density of Olig2^+^ cells (Fig. 5F, G), Pdgfrα^+^/Olig2^+^ cells (Fig. 5F, H), or CC1^+^/Olig2^+^ cells (Fig. 5F, I) in the cortex of *Tmem63a*^*−/−*^ mice compared to WTs. The above results suggested that myelin abnormalities, though partially recovered, persist in the cortex of *Tmem63a*^*−/−*^ mice at P21.

Western blot analysis on corpus callosum samples from P21 mice (Fig. S4A) showed no significant differences in the levels of Mag and Mbp between control and *Tmem63a*^*−/−*^ mice (Fig. S4B, C). IF of Mag and Mbp on brain sections at P21 in the corpus callosum of *Tmem63a*^*−/−*^ mice were comparable to that of the controls (Fig. S4D). TrueGold myelin staining showed there was no significant difference in the density of TrueGold-positive signals in the corpus callosum of *Tmem63a*^*−/−*^ mice compared with WTs (Fig. S4E). Finally, there was no difference in the density of Olig2^+^ cells (Fig. S4F, G), Pdgfrα^+^/Olig2^+^ cells (Fig. S4F, H), and CC1^+^/Olig2^+^ cells (Fig. S4F, I) in the corpus callosum. These data suggested that the myelin is recovered in the corpus callosum by P21.

**Fig. 6:**
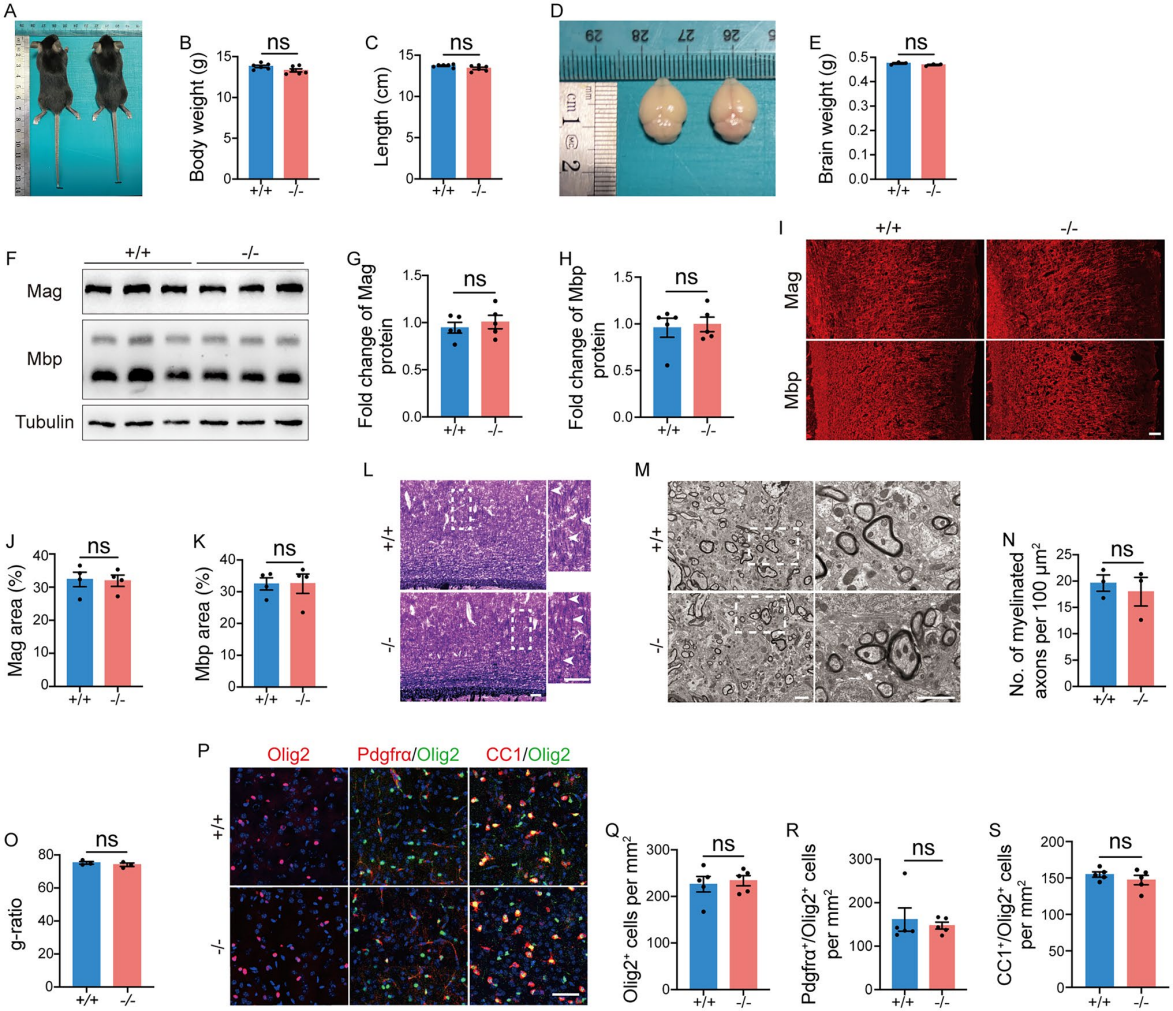
Myelin in *Tmem63a*^*−/−*^ mice is normal at P28. **A–C** Body size of WT and *Tmem63a*^*−/−*^ mice. The body weight (**B**) (*P =* 0.0689, *t*-test) and the length of the body (**C**) (*P =* 0.0825, *t*-test) are not significantly different between *Tmem63a*^*−/−*^ mice and WTs at P28 (*n =* 6 mice per group). **D** Whole brains of WT and *Tmem63a*^*−/−*^ mice at P28. **E** Quantitative analysis showing no significant difference in the size of the brain between *Tmem63a*^*−/−*^ mice and WTs at P28 (*P* = 0.2058, *t*-test; *n =* 3 mice per group).** F** Western blotting for Mag and Mbp. Lysates are prepared from the cortex in WT and *Tmem63a*^*−/−*^ mice at P28. β-Tubulin is used as the loading control. **G, H** Fold change of protein levels. There are no significant differences in Mag (**G**) (*P* = 0.5235, *t*-test) and Mbp (**H**) (*P* = 0.7869, *t*-test) between WT and *Tmem63a*^*−/−*^ mice (*n* = 5 mice per group). **I** Representative images of IF for Mag and Mbp. Scale bar, 100 μm. **J, K** Ratio of the immuno-reactivity area to total area. The immuno-reactivity for Mag (**J**) (*P* = 0.8880, *t*-test) or Mbp (**K**) (*P* = 0.9829, *t*-test) does not significantly differ between control and *Tmem63a*^*−/−*^ mice at P28 (*n* = 4 mice per group). **L** TrueGold myelin staining of sections from WT and *Tmem63a*^*−/−*^ mice at P28. The panels on the right are enlarged from the boxed areas. Scale bars, 100 μm. **M** Electron microscopic images and analysis of the myelin sheath. The panels on the right are enlarged from the boxed areas. Scale bars, 2 μm. **N** Numbers of myelinated axons per 100 μm^2^ are comparable between control and *Tmem63a*^*−/−*^ mice (*P* = 0.6305, *t*-test; *n* = 3 mice per group). **O** Myelin sheath thickness and bar graph of *g*-ratios of all axons as a function of axonal diameter. The *g*-ratio is comparable in control and *Tmem63a*^*−/−*^ mice (*P* = 0.4370, *t*-test; *n* = 3 mice per group). **P** Representative images of IF for Olig2, Pdgfrα/Olig2, and CC1/Olig2 in the cortex at P28. Scale bar, 50 μm. **Q** The density of Olig2^+^ cells in the cortex (*P* = 0.7151, *t*-test; *n* = 5 mice per group). **R** The density of Pdgfrα^+^/Olig2^+^ cells in the cortex (*P* = 0.6285, *t*-test, *n* = 5 mice per group). **S** The density of CC1^+^/Olig2^+^ cells in the cortex (*P* = 0.3369, *t*-test; *n* = 5 mice per group). ns, not significant.

By P28, there were no significant differences in body weight (Fig. 6A, B) and body length (Fig. 6A, C) between *Tmem63a*^*−/−*^ mice and WTs. Meanwhile, the brain weights of *Tmem63a*^*−/−*^ and WT mice were compared (Fig. 6D, E). We subsequently assessed the expression of myelin proteins in *Tmem63a*^*−/−*^ mice in comparison to controls at P28. Western blot analysis was applied to cortical samples from P28 mice (Fig. 6F), revealing no significant differences in the levels of Mag and Mbp (Fig. 6G, H). We then applied IF to brain sections at P28 (Fig. 6I), which indicated that the immunoreactivity of Mag and Mbp in *Tmem63a*^*−/−*^ mice was comparable to that of the controls (Fig. 6J, K). TrueGold myelin staining showed there was no significant difference in the density of TrueGold-positive signals between *Tmem63a*^*−/−*^ mice and WTs (Fig. 6L). Further, TEM demonstrated that the density of myelinated axons (Fig. 6M, N) and the thickness of the myelin sheath (Fig. 6M, O) were similar in control and *Tmem63a*^*−/−*^ mice at P28. Finally, we applied IF to OL lineage cells using several markers at P28 (Fig. 6P). There was no difference in the density of Olig2^+^ cells (Fig. 6Q), Pdgfrα^+^/Olig2 ^+^ cells (Fig. 6R), or CC1^+^/Olig2 ^+^ cells (Fig. 6S) in the cortex between WTs and *Tmem63a*^*−/−*^ mice. Collectively, these results indicate that, although the expression of myelin proteins was significantly reduced at P14, it had fully recovered by P28. These findings suggest that the TMEM63A protein may be a critical regulator that determines the timing of OL differentiation during development.

### Myelin Dysplasia in Primary Cultured ***Tmem63a***^***−/−***^ OPCs

**Fig. 7:**
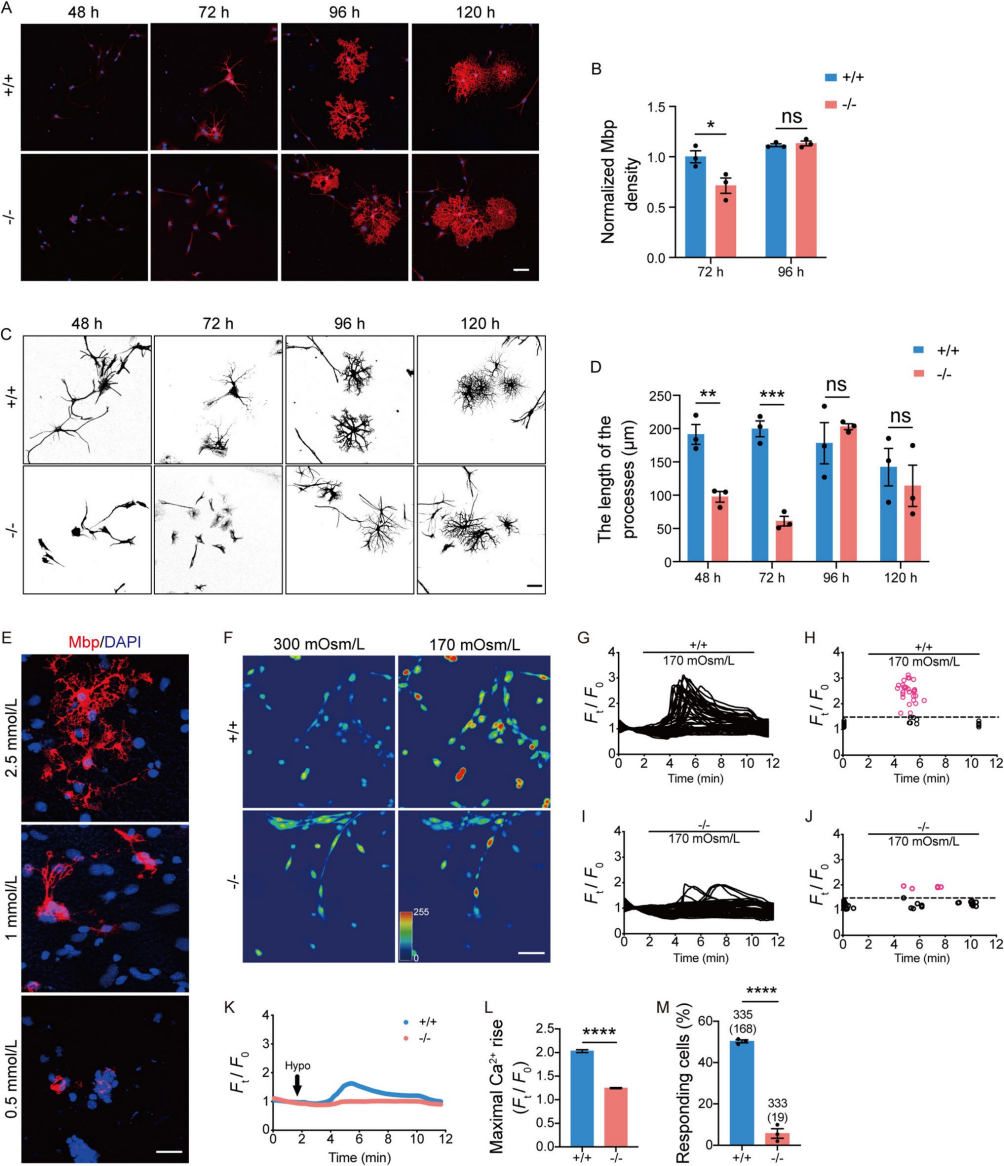
Myelin dysplasia in primary cultured *Tmem63a*^*−/−*^ OPCs. **A** Immunolabeling for Mbp in primary OPCs differentiated for 48, 72, 96, or 120 h. Scale bar, 50 μm. **B** Normalized Mbp density is significantly lower in *Tmem63a*^*−/−*^ OPCs than controls differentiated for 72 h (72 h: *P* = 0.0413, *t*-test; 96 h: *P* = 0.5864, *t*-test; *n* = 3 mice per group). **C** Representative OLs in primary OPCs differentiated for 48, 72, 96, or 120 h illustrated with the method of Sholl analysis. Scale bar, 50 μm. **D** Sholl analysis of average process length per cell (48 h: *P* = 0.0052, *t*-test; 72 h: *P* = 0.0006, *t*-test; 96 h: *P* = 0.4697, *t*-test; 120 h: *P* = 0.5408, *t*-test; *n* = 3 mice per group). **E** Immunolabeling for Mbp in primary OPCs under differentiation conditions containing different concentrations of Ca^2+^ for 72 h. Scale bar, 50 μm. **F** Fluorescent emission images of OLs differentiated for 20 h, in response to hypotonic stimulation. Scale bar, 100 μm. **G** Ca^2+^ fluorescence intensity traces in WT OLs respond to 170 mOsm/L solution in a representative experiment. **H** The maximal responses of cells pooled from three experiments challenged by 170 mOsm/L solution *versus* exposure time. Red, Ca^2+^ fluorescence increases >50% are considered to be positively-responding cells. **I** Ca^2+^ fluorescence intensity traces in *Tmem63a*^*−/−*^ OLs responding to 170 mOsm/L solution in a representative experiment. **J** The maximal responses of cells pooled from three experiments challenged by 170 mOsm/L solution *versus* exposure time. Red, Ca^2+^ fluorescence increases >50% are considered to be positively-responding cells. **K** Ca^2+^ intensity traces of WT and *Tmem63a*^*−/−*^ OLs respond to 170 mOsm/L solution in a representative experiment. **L** The maximal Ca^2+^ rise in OLs in response to hypotonic stimulation (****P <0.0001, *t*-test; +/+: *n* = 335 cells; −/−: *n* = 333 cells). **M** Percentage of cells responding to hypotonic stimulation under indicated extracellular [Ca^2+^] from three experiments. The number of cells tested and responding (in brackets) is indicated above the bars (****P <0.0001, *t*-test; *n* = 3 mice per group). *P <0.05, **P <0.01, ***P <0.001; ns, not significant.

In order to further validate the above findings, OPCs were prepared from the cortex of P7 pups and cultured in an oligosphere medium. After 3 days, a mitogen-free medium was applied to promote the differentiation of OPCs. Generally, after culture in the mitogen-free medium for 48 h, processes were extended from the cell body, suggesting that OPCs were differentiating into OLs. The processes then extended and branched, and the OLs became increasingly complex in arborization *in vitro*. The morphology of OLs (process formation and branching) and the expression of myelin proteins were analyzed after differentiation for 48, 72, 96, and 120 h *in vitro*. The fluorescence immunohistochemistry of Mbp was significantly lower in differentiated OLs from *Tmem63a*^*−/−*^ mice than those from WTs at 48 or 72 h but did not differ at 96 and 120 h (Fig. 7A, B). Besides, the average length of the processes of OLs from *Tmem63a*^*−/−*^ mice was significantly shorter than that of the WT OLs at 48 or 72 h but comparable at 96 and 120 h (Fig. 7C, D). These results suggested that the myelin dysplasia caused by *Tmem63a* deletion is temporary.

### TMEM63A Induces Ca^2+^ Influx in OPC/OL Cultures

TMEM63 proteins function as osmosensitive cation channels that regulate extracellular Ca^2+^ influx, and previous research has indicated that TMEM63A, in particular, acts as an osmosensitive cation channel activated by hypotonic stress, mediating extracellular Ca^2+^ influx [14]. This prompted us to hypothesize that TMEM63A-mediated Ca^2+^ influx plays a pivotal role in OL differentiation. To test this possibility, we manipulated the Ca^2+^ concentration in the OL differentiation medium. Notably, we found Mbp^+^ cells at 72 h after differentiation in mitogen-free medium treated with 2.5 mmol/L Ca^2+^ but not 0.5 mmol/L Ca^2+^ (Fig. 7E), underscoring the importance of Ca^2+^ influx in OL differentiation.

To further characterize the ionotropic action of TMEM63A in OLs, we cultured OPCs isolated from WT and *Tmem63a*^−/−^ mice in mitogen-free medium for 20 h. These cells were then loaded with the Ca^2+^ indicator dye Fluo-4-AM, and changes in cytoplasmic [Ca^2+^]_i_ were monitored after switching the extracellular osmolarity from 300 to 170 mOsm/L (Fig. 7F). This hypotonic stimulus induced a ~1.6-fold elevation in Ca^2+^ fluorescence from the isotonic baseline in WT OLs, while *Tmem63a*^−/−^ OLs exhibited less elevation (Fig. 7G–L). Furthermore, the ratio of responding cells among *Tmem63a*^−/−^ OLs was significantly lower than the control (Fig. 7M). These results support the conclusion that TMEM63A is a key regulator of Ca^2+^ influx in OLs during the early stages of OPC differentiation.

### TMEM63A_A632T Fails to Rescue Myelination

**Fig. 8:**
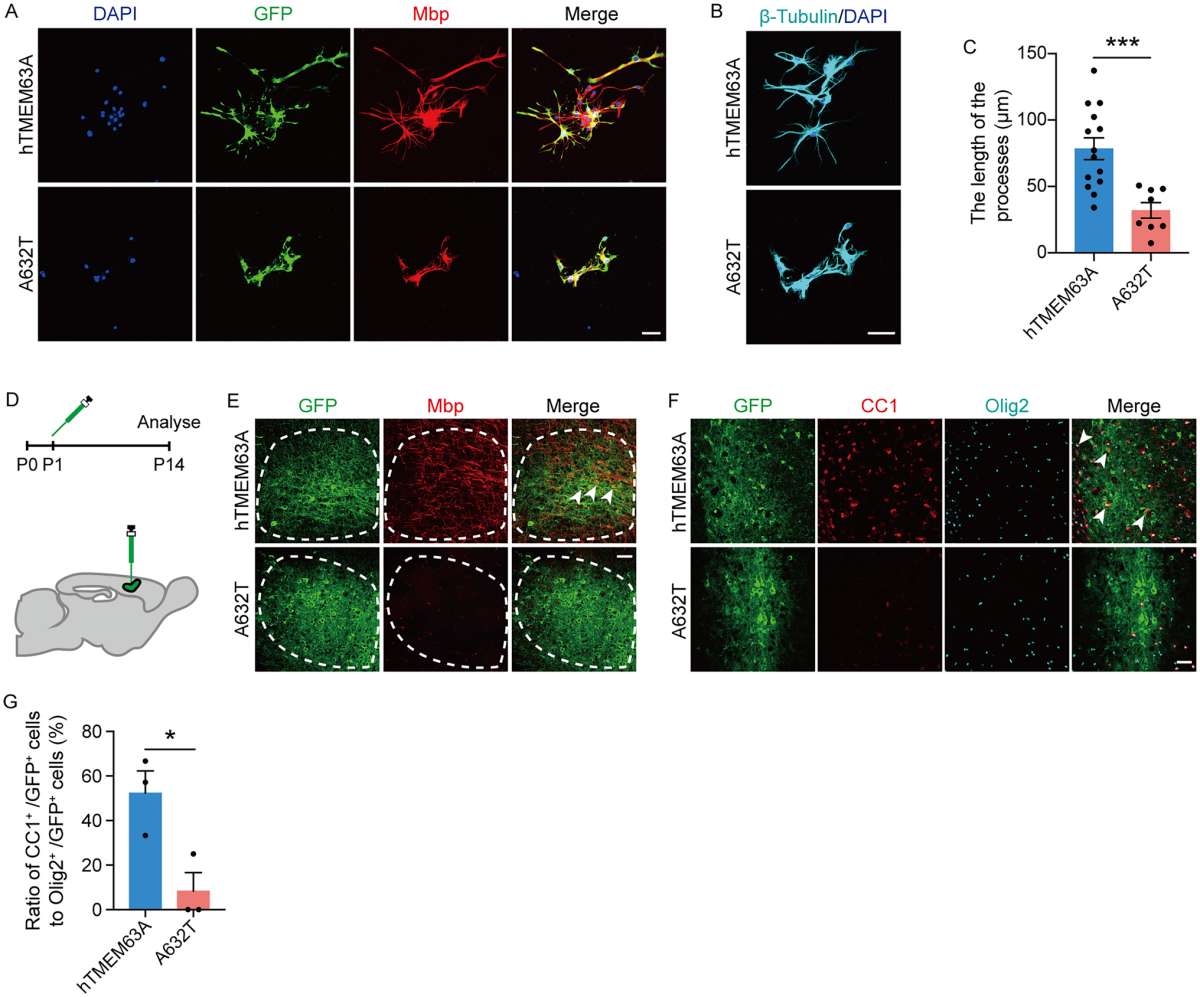
TMEM63A_A632T fails to rescue myelination. **A** Double-staining of OLs for Mbp and GFP. Scale bar, 50 μm. **B** Representative images of β-tubulin immunofluorescence in oligodendrocyte cultures at 3 days *in vitro* (DIV3). Scale bar, 50 μm. **C** Average sheath length per cell. OLs with TMEM63A_A632T show a shorter branch length than WTs (*P* = 0.0008, *t*-test; WT, *n* = 14; A632T, *n* = 8). **D** Experimental paradigm for lentivirus injection in the cortex of *Tmem6a*^*−/−*^ mice. **E** Representative images of Mbp and GFP immunofluorescence. The immuno-reactivity for Mbp is extremely low in the cortex in the *Tmem63a*^*−/−*^ mice re-expressing TMEM63A_A632T at P14. The white arrowheads indicate the double positive signaling. Scale bar, 50 μm. **F** Representative images for immunofluorescence on GFP, Olig2, and CC1. The white arrowheads indicate the GFP^+^/CC1^+^/Olig2^+^ cells. Scale bar, 50 μm. **G** The ratio of CC1^+^/GFP^+^ cells to Olig2^+^/GFP^+^ is extremely low in the cortex of the *Tmem63a*^*−/−*^ mice re-expressing TMEM63A_A632T at P14 (*P* = 0.0272, *t*-test; *n* = 3 mice per group). *P <0.05, ***P <0.001.

We then explored whether expressing human TMEM63A in *Tmem63a*^−/−^ OPCs rescues their differentiation. Thus, we constructed lentivirus (LV) carrying human TMEM63A (LV-TMEM63A) and infected cultured *Tmem63a*^−/−^ OPCs. Significant OL differentiation was seen in OPCs infected with LV-TMEM63A cultured for 72 h in a mitogen-free medium (Fig. 8A), in sharp contrast to uninfected OPCs. Conversely, OPCs infected with LV-TMEM63A_A632T showed very limited differentiation by 72 h in a differentiation medium (Fig. 8A). The processes were longer in cells infected with LV-TMEM63A than those with LV-TMEM63A_A632T (Fig. 8B, C). Furthermore, we microinjected LV-TMEM63A into the cortex of *Tmem63a*^−/−^ mice at P1 (Fig. 8D) and examined the expression of Mbp in the cortex at P14. Immunostaining showed that the immunoreactivity of Mbp was significantly weaker in the GFP fluorescence region of the cortex in mice with LV-TMEM63A_A632T compared to those infected with LV-TMEM63A (Fig. 8E). The density of GFP^+^/Olig2^+^/CC1^+^ cells was also significantly lower (Fig. 8F, G). These data indicate that re-expressing TMEM63A in *Tmem63a*^−/−^ OLs can rescue their differentiation. The rescue effects of the LoF mutant A632T are much weaker.

### ***Tmem63a***^***+/−***^ Mice Exhibit Normal Myelination

To date, hypomyelination-related *TMEM63A* mutant patients including ours are all heterozygous *de novo* mutations. Thus, it is of interest to know whether *Tmem63a*^*+/−*^ mice could have hypomyelination phenocopying these patients. We thus examined myelin-related phenotypes in the *Tmem63a*^*+/−*^ mice. The protein levels of Mag and Mbp were comparable in the cortex between control and *Tmem63a*^*+/−*^ mice at P14 (Fig. S5A–C). Furthermore, Mag^+^ and Mbp^+^ myelin were unaltered in the *Tmem63a*^*+/−*^ mice (Fig. S5D-F). TrueGold myelin staining showed no significant difference between WT and *Tmem63a*^*+/−*^ mice (Fig. S5G). Overall, these observations suggest that the myelination appears normal in the *Tmem63a*^*+/−*^ mice. These results indicate that *Tmem63a* genetic dose insufficiency may be more severe in humans than in mice. Alternatively, the mutant *TMEM63A* may interfere with the healthy copy of *TMEM63A* in the patients.

## Discussion

During development, OPCs undergo drastic changes in their morphology, and the process of myelination occurs with precise timing and spatial coordination [6]. It is proposed that an internal clock exists to measure the progression of OL development [56]. This process requires precise regulation of a series of intracellular and extracellular factors. The TMEM63A channel is one such factor that was recently revealed to be involved in early myelin sheath development in humans. Missense mutations in the *TMEM63A* gene result in hypomyelination leukodystrophy, which displays congenital nystagmus and deficient myelination in the brain of infants [29–31]. However, there is still a lack of animal models to validate this genotype-phenotype association. The underlying mechanism of how TMEM63A regulates myelination remains to be established.

In this study, we developed *Tmem63a*^*EGFP*^ knockin and *Tmem63a*^*−/−*^ mice. The *Tmem63a*^*EGFP*^ line expresses EGFP driven by the endogenous *Tmem63a* promoter, thus the distribution of EGFP protein represents TMEM63A expression. We found that the EGFP signal mostly (~90%) co-stained with Olig2, suggesting that TMEM63A channels are mostly expressed in the OL lineage. The *Tmem63a*^−/−^ mice exhibited temporary hypomyelination in the cortex at ~2 weeks, which fully recovered by P28. The hypomyelination in the corpus callosum is recovered earlier by P21. These observations phenocopied the symptoms in human patients, that TMEM63A mutations cause hypomyelination in infants but later recover [29–31]. Interestingly, in primary *Tmem63a*^−/−^ OPC culture, induction of differentiation causes a temporary deficiency in OL differentiation. Virus-based expression of TMEM63A promoted the differentiation of cultured *Tmem63a*^−/−^ OPCs. These results suggest that TMEM63A regulates myelination in a cell-autonomous manner.

Ca^2+^ plays a critical role in the development of OLs, including migration, differentiation, maturation, and myelin sheath growth [57–59]. There are several potential routes of Ca^2+^ influx across the OL plasma membrane, including direct influx through voltage- or ligand-gated channels [60]. Our study indicates that Ca^2+^ entry through TMEM63A channels in OLs significantly contributes to the Ca^2+^ signal in the early stage of OL differentiation.

The EGFP expression in the developmental brains of *Tmem63a*^*EGFP*^ knockin mice suggests that TMEM63A increases with OL differentiation and maturation. Why does TMEM63A deletion only affect the early development of OLs? We speculate that two possibilities may account for this phenotype. First, in *Tmem63a* KO mice, the *Tmem63a* gene is inactivated from the earliest development stage. Compensatory mechanisms may be triggered during development to alleviate the loss of the *Tmem63a* gene. Another possibility may be due to functional redundancy. In *Tmem63a* KO mice, other mechanisms could contribute to the late phase of OL development. In our observation, the myelination recovers to a normal level after P28. Could TMEM63A play a role in OLs in adults? It is of interest, we believe, to know whether TMEM63A regulates remyelination when myelin is damaged in the brain. The *Tmem63a* deficient mice generated in this study could help to address this question in the future.

In this study, we found that the *Tmem63a *^*−/−*^ mice generally exhibited smaller body weight and brain sizes compared with WT at P14 but comparable at P28. The development of myelin sheath and the changes in body weight and brain size were somewhat synchronized, indicating there might be some sort of linkages among them in the *Tmem63a*^*−/−*^ mice. However, these data do not exclude the possibility that the changes in body weight could be related to TMEM63A function in other organs but not the brain.

We also identified a rare variant, A632T, in the *TMEM63A* gene in a child with developmental delay and hypomyelination. Ca^2+^ imaging assays *in vitro* revealed that TMEM63A_A632T is a LoF mutation. Virus-based expression of TMEM63A_A632T in primary *Tmem63a*^−/−^ OPCs failed to rescue OL differentiation. Microinjection into the cortex also failed to rescue myelination defects. These results strongly suggested that the mutation in TMEM63A is most likely the pathogenetic factor for the patients. According to previous reports that TMEM63A mutations cause temporary developmental delay, we would optimistically predict that the child will recover at some point.

In summary, we have demonstrated that TMEM63A plays a critical role at the functional level in oligodendrocyte differentiation both *in vivo* and *in vitro* during an early and precise time window. The finding that Ca^2+^ influx mediated by TMEM63A in oligodendrocytes may be critical for normal postnatal myelination significantly enhances our comprehension of the regulatory network underlying myelin development. Moreover, it highlights the potential of TMEM63A as a promising therapeutic target for disease intervention.

## Supplementary Information

Below is the link to the electronic supplementary material.

Supplementary file1 (PDF 1082 kb)

## Data Availability

All data are available upon request to the corresponding authors.
