# Supplementary material for: Cation Channel TMEM63A Autonomously Facilitates Oligodendrocyte Differentiation at an Early Stage
Source: Neurosci Bull. 2025 Feb 21;41(4):615–32. doi: 10.1007/s12264-024-01338-4 (PMC11978589; doi:10.1007/s12264-024-01338-4)
Supplement: Supplementary file 1 — Supplementary file1 (PDF 1082 kb) [file 12264_2024_1338_MOESM1_ESM.pdf]

Supplementary Materials

Supplementary Figures

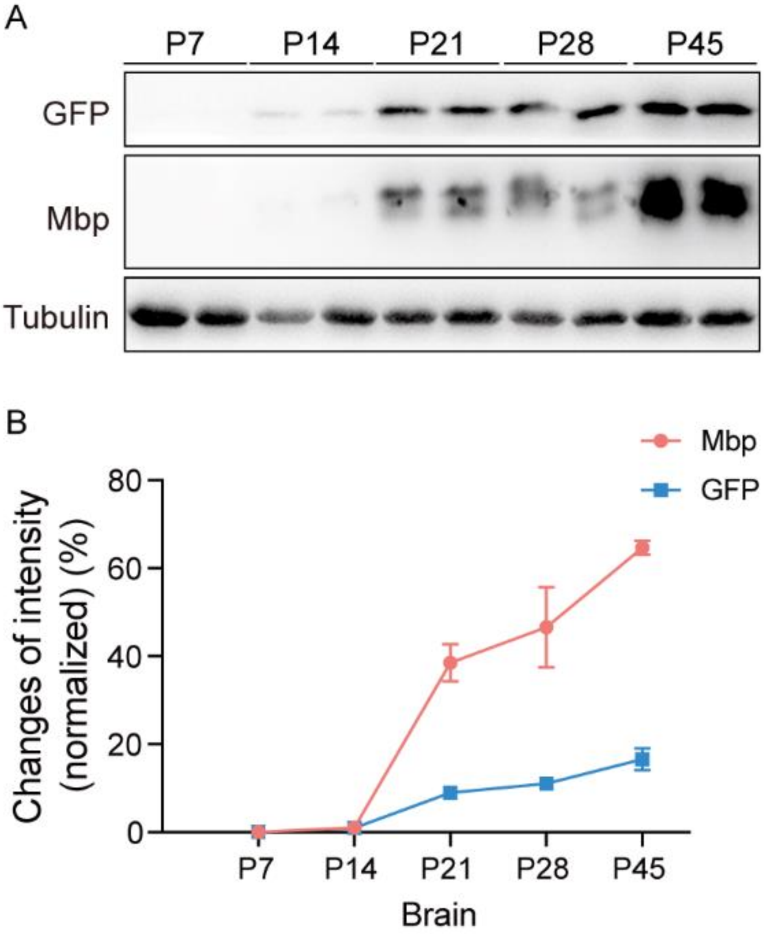

Fig. S1 Correlated expression between GFP and Mbp in the brain of *Tmem63a*$^{EGFP/+}$ mice. **A** The expressions of TMEM63A and Mbp in the brain from postnatal mice. **B** Graph showing Mbp and GFP expression levels (4 mice at each time point).

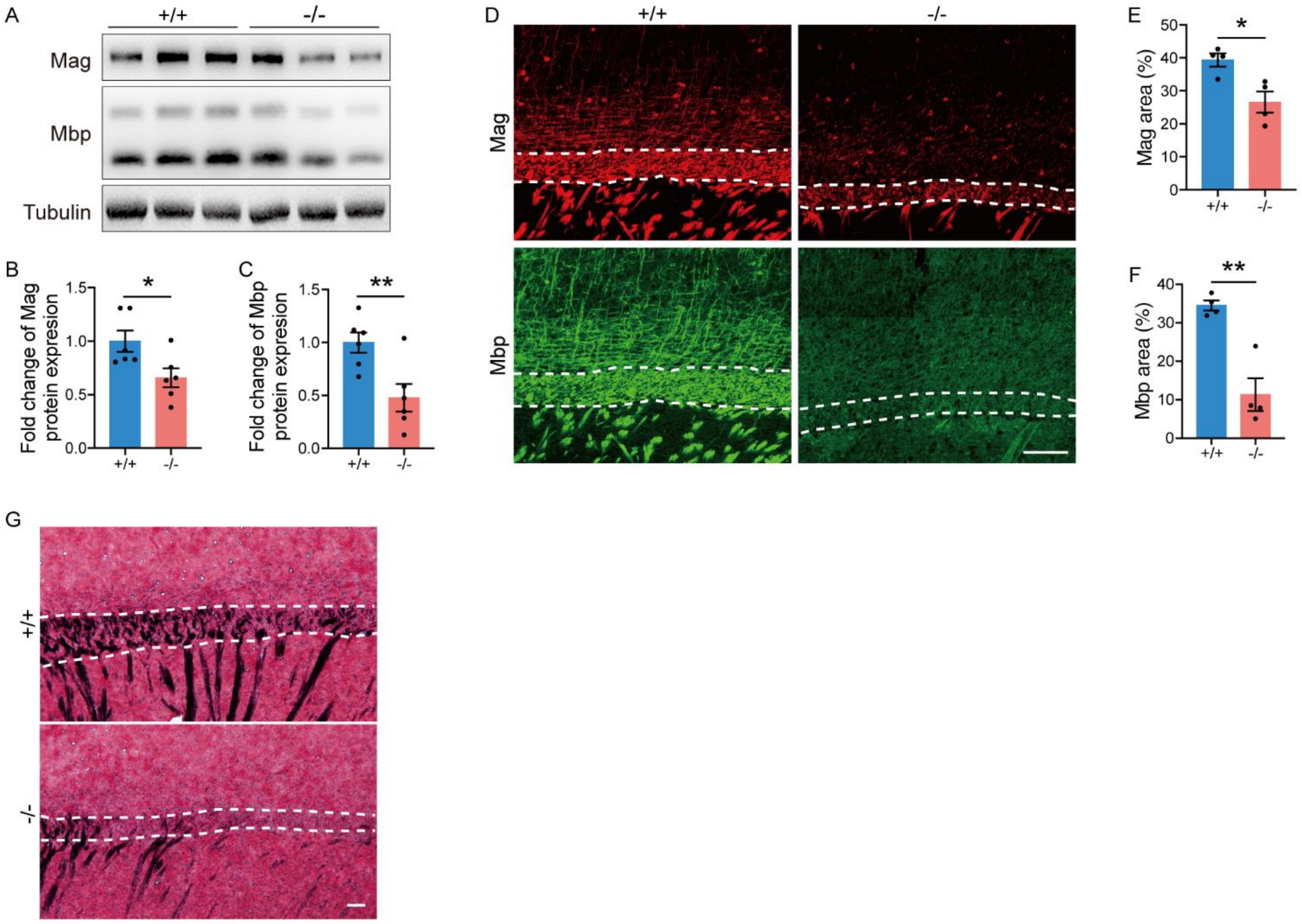

Fig. S2 Loss of TMEM63A disrupts proper myelination in the corpus callosum. **A** Western blotting for Mag and Mbp. Lysates were prepared from the corpus callosum in WT and *Tmem63a*$^{-/-}$ mice at P14. β-Tubulin was used as the loading control. **B, C** Fold change of protein levels. Mag (**B**) (**P* = 0.0272, *t*-test) and Mbp (**C**) (***P* = 0.0089, *t*-test) between WT and *Tmem63a*$^{-/-}$ mice (*n* = 6 mice per group). **D** Representative images for fluorescence IHC on Mag and Mbp. Scale bar, 100 µm. **E, F** Ratio of the immuno-reactivity area to total area on Mag (**E**) (**P* = 0.0152, *t*-test) or Mbp (**F**) (***P* = 0.0020, *t*-test) in the corpus callosum of the WT and *Tmem63a*$^{-/-}$ mice at P14 (*n* = 4 mice per group). **G** TrueGold myelin staining. Brain sections from WT and *Tmem63a*$^{-/-}$ mice at P14 were used. Scale bar, 50 µm.

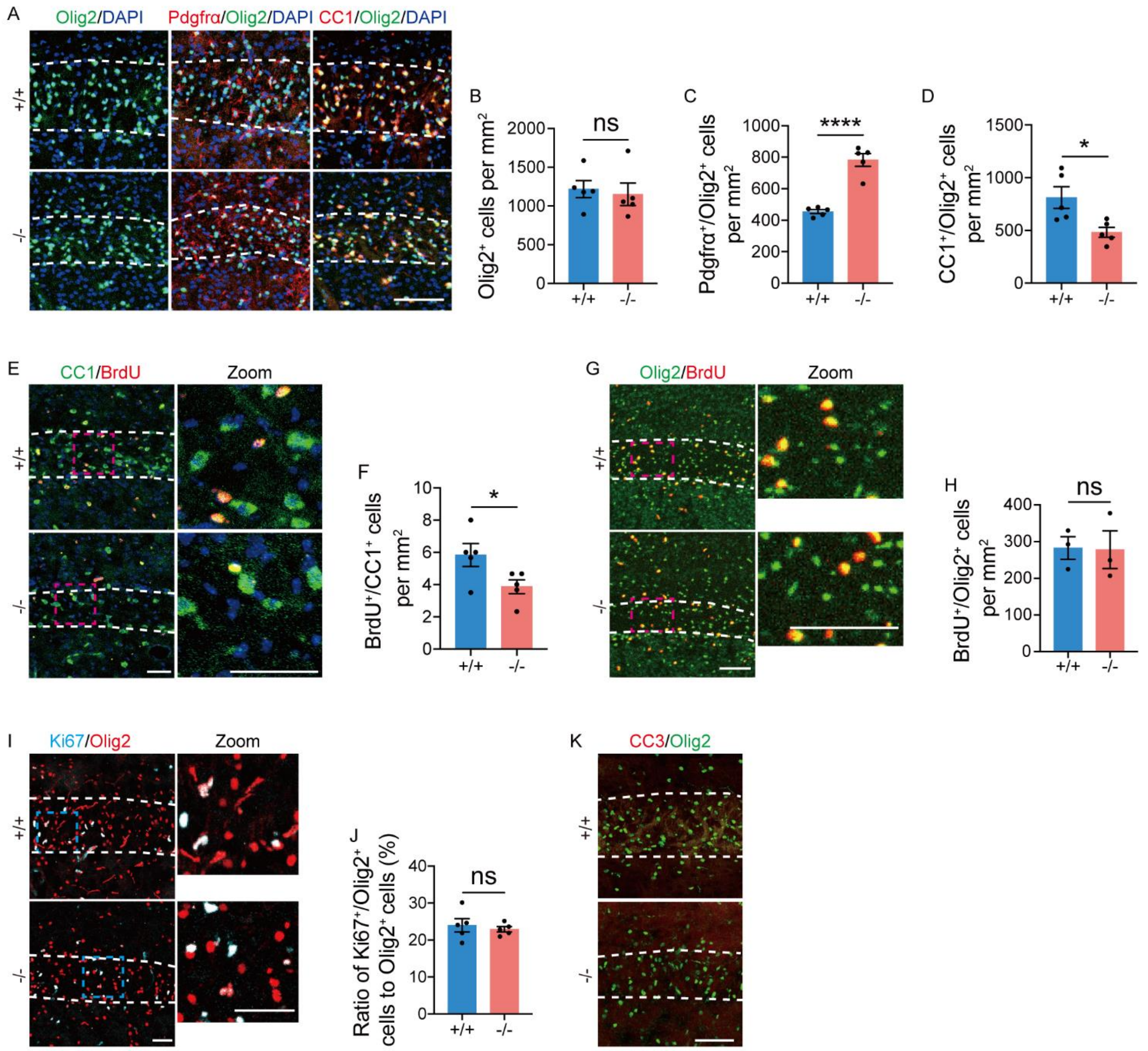

Fig. S3 Loss of TMEM63A impedes oligodendrocyte differentiation in the corpus callosum. **A** Representative images for IF on Olig2, Pdgfrα/Olig2, and CC1/Olig2 taken from the corpus callosum of control or *Tmem63a*$^{-/-}$ mice at P14. **B** The density of Olig2^{+} cells in the corpus callosum (*ns*. P = 0.7276, t-test; n = 5 mice per group). **C** The density of Pdgfrα^{+}/Olig2^{+} cells in the corpus callosum (****P <0.0001, t-test; n = 5 mice per group). **D** The density of CC1^{+}/Olig2^{+} cells in the corpus callosum (*P = 0.0191, t-test; n = 5 mice per group). **E** Representative images of double-staining for BrdU/CC1 taken from the corpus callosum of control or *Tmem63a*$^{-/-}$ mice at P14. The panels on the right are enlarged from boxed areas. **F** The number of BrdU^{+}/CC1^{+} cells per mm^2 in the corpus callosum (*P = 0.0461, t-test; n = 5 mice per group). **G** Representative images of double-staining for Olig2/BrdU taken from the corpus callosum of control or *Tmem63a*$^{-/-}$ mice at P4. The panels on the

right are enlarged from boxed areas. **H** The number of BrdU$^+$/Olig2$^+$ cells in the corpus callosum (*ns*. P = 0.9424, *t*-test; n = 3 mice per group). **I** Representative images of double-staining for Olig2/Ki67 in the corpus callosum. Brain sections were used from control and *Tmem63a*$^{-/-}$ mice at P14. The panels on the right are enlarged from boxed areas. **J** Ratio of Ki67$^+$/Olig2$^+$ cells to Olig2$^+$ cells (*ns*. P = 0.5866, *t*-test; n = 5 mice per group). **K** Representative images of double-staining for CC3/Olig2 in the corpus callosum. Brain sections were used from control and *Tmem63a*$^{-/-}$ mice at P14. The scale bars are 100 μm in **A**, **G**, and **K**, and 50 μm in **E** and **I**.

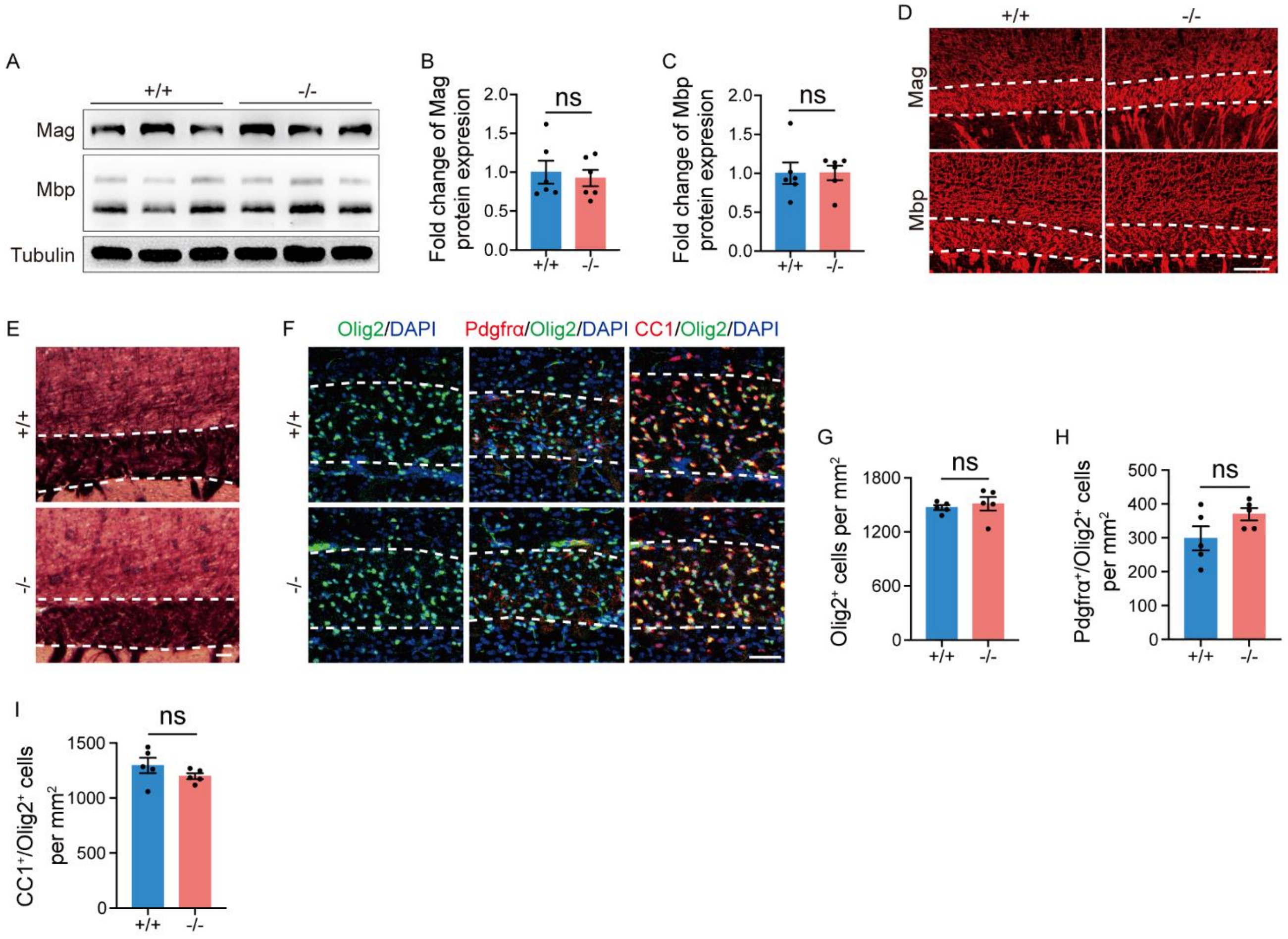

Fig. S4 Myelin in *Tmem63a*$^{-/-}$ mice is normal at P21 in the corpus callosum. **A** Western blotting for Mag and Mbp. Lysates were prepared from the corpus callosum in WT and *Tmem63a*$^{-/-}$ mice at P21. β-Tubulin was used as the loading control. **B, C** Fold change of protein levels. There are no significant differences in Mag (**B**) (*ns*. $P = 0.6833$, *t*-test) and Mbp (**C**) (*ns*. $P = 0.9761$, *t*-test) between WT and *Tmem63a*$^{-/-}$ mice ($n = 6$ mice per group). **D** Representative images for IF on Mag and Mbp. Scale bar, 100 µm. **E** TrueGold myelin staining. Brain sections from WT and *Tmem63a*$^{-/-}$ mice at P21 were used. The myelin sheath tracts in the corpus callosum are not significantly different between control and *Tmem63a*$^{-/-}$ mice at P21. Scale bar, 200 µm. **F** Representative images for IF on Olig2, Pdgfrα/Olig2, and CC1/Olig2 in the corpus callosum at P21. Scale bar, 50 µm. **G** The density of Olig2^{+} cells in the corpus callosum (*ns*. $P = 0.6210$, *t*-test; $n = 5$ mice per group). **H** The number of Pdgfrα^{+}/Olig2^{+} cells in the corpus callosum (*ns*. $P = 0.1121$, *t*-test; $n = 5$ mice per group). **I** The number of CC1^{+}/Olig2^{+} cells in the corpus callosum (*ns*. $P = 0.2289$, *t*-test; $n = 5$ mice per group).

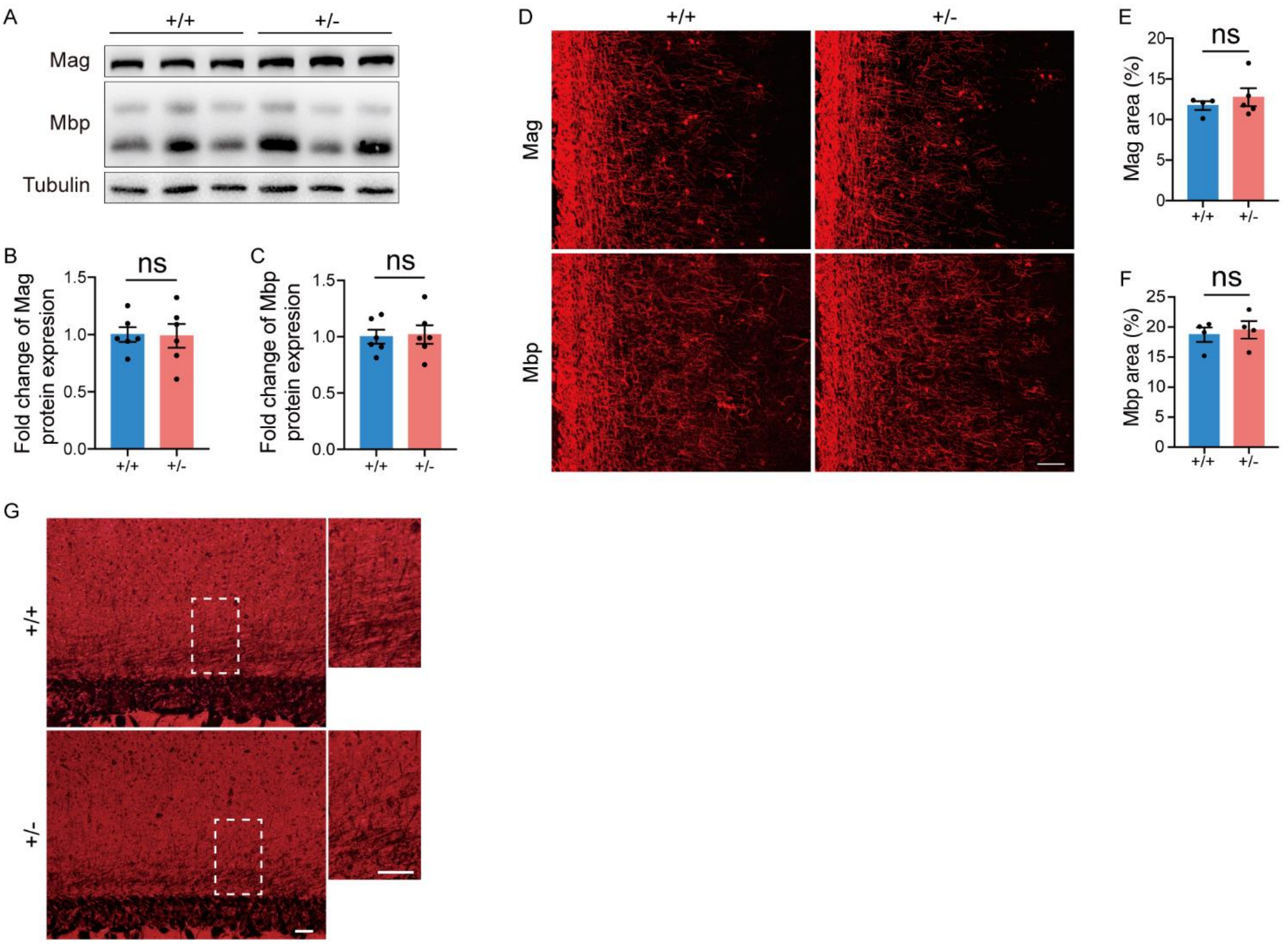

Fig. S5 Myelin was normal in the *Tmem63a*$^{+/-}$ mice. **A** Western blotting for Mag and Mbp. Lysates were prepared from the cortex in WT and *Tmem63a*$^{+/-}$ mice at P14. β-Tubulin was used as the loading control. **B, C** Fold change of protein levels. Mag (**B**) (*ns*. P = 0.9243, t-test) and Mbp (**C**) (*ns*. P = 0.8556, t-test) between WT and *Tmem63a*$^{+/-}$ mice (n = 6 mice per group). **D** Representative images for IF on Mag and Mbp. Scale bar, 100 μm. **E, F** Ratio of the immuno-reactivity area to total area on Mag (**E**) (*ns*. P = 0.4765, t-test; WT: n = 4 mice, *Tmem63a*$^{+/-}$: n = 5 mice) or Mbp (**F**) (*ns*. P = 0.6878, t-test; n = 4 mice per group) between control and *Tmem63a*$^{+/-}$ mice at P14. **G** TrueGold myelin staining. Brain sections from WT and *Tmem63a*$^{+/-}$ mice at P14 were used. Scale bars, 50 μm.
